# Oxidation and alkylation stresses activate ribosome-quality control

**DOI:** 10.1038/s41467-019-13579-3

**Published:** 2019-12-09

**Authors:** Liewei L. Yan, Carrie L. Simms, Fionn McLoughlin, Richard D. Vierstra, Hani S. Zaher

**Affiliations:** Department of Biology, Washington University in St. Louis, St. Louis, MO 63130 USA

**Keywords:** RNA, RNA quality control

## Abstract

Oxidation and alkylation of nucleobases are known to disrupt their base-pairing properties within RNA. It is, however, unclear whether organisms have evolved general mechanism(s) to deal with this damage. Here we show that the mRNA-surveillance pathway of no-go decay and the associated ribosome-quality control are activated in response to nucleobase alkylation and oxidation. Our findings reveal that these processes are important for clearing chemically modified mRNA and the resulting aberrant-protein products. In the absence of Xrn1, the level of damaged mRNA significantly increases. Furthermore, deletion of LTN1 results in the accumulation of protein aggregates in the presence of oxidizing and alkylating agents. This accumulation is accompanied by Hel2-dependent regulatory ubiquitylation of ribosomal proteins. Collectively, our data highlight the burden of chemically damaged mRNA on cellular homeostasis and suggest that organisms evolved mechanisms to counter their accumulation.

## Abstract (web summary)

The mRNA-surveillance pathway of no-go decay (NGD) is a eukaryotic ribosome-based-quality-control process that targets transcripts that stall the ribosome. Here the authors show no-go decay (NGD) and ribosome-quality control (RQC) pathways are activated by mRNAs damaged by alkylation and oxidation stress.

## Introduction

The mRNA-surveillance pathway of no-go decay (NGD) is a eukaryotic ribosome-based, quality-control mechanism that targets transcripts stalled on ribosomes during translation^1,2^. Much of the mechanistic details of NGD have emerged from a wealth of studies using yeast as the model^1–5^, but the process appears to be highly conserved and employs a homologous set of factors in most other eukaryotes. In particular, when ribosomes stall upon encountering a defective mRNA, three key events occur: (i) the defective transcript is degraded rapidly; (ii) the stalled ribosomes are rescued; and (iii) the incomplete-nascent peptides are subjected to proteolysis. RNA breakdown is accomplished by at least two pathways; a primary route that involves the major 5′–3′-exonuclease Xrn1 and a secondary route triggered through an endonucleolytic cleavage of the bound transcript upstream of the stalled ribosome^1,2,4–10^. Endonucleolytic cleavage generates an uncapped 3′-fragment and a deadenylated 5′-fragment, which are rapidly turned over through the exonucleolytic actions of Xrn1 and the exosome, respectively. As a result of this cleavage, ribosomes translating the 5′-fragment run to the end of the mRNA, hence displaying an empty A site, which in turn marks them for the dissociating activity of Dom34/Hbs1/Rli1^4,5,7^. The splitting action of Dom34/Hbs1/Rli1 leaves a peptidyl-tRNA-bound large ribosomal subunit that is recognized by the ribosome-associated-quality-control (RQC) process^8,9^. RQC engages a number of factors to recognize the incomplete-nascent peptide, release it from stalled ribosomes, and deliver it to the proteasome for breakdown. The ubiquitin-protein ligase (E3) Ltn1 has a key role during this process by adding K48-linked polyubiquitin chains to the nascent peptide while bound to the 60S subunit as a peptidyl-tRNA adduct^6,8^. Following release of the ubiquitylated peptide by the nuclease Vms1^11–15^, the free species is recognized by the Cdc48-Ufd1-Npl4 disaggregase complex for final presentation to the proteasome^9,11,16,17^.

Although NGD and RQC are initiated by the same signal—a stalled ribosome—how activation of the two processes is coordinated and, more specifically, how quality-control factors recognize and are recruited to ribosomes stalled on aberrant mRNAs remained largely unclear until recently. The discovery that ribosomal proteins are ubiquitylated during stalling and that the trigger for this modification is collided ribosomes shed new light on the activation events surrounding NGD and RQC^18–21^. In yeast, the E3 ligase Hel2 has been shown to be a critical effector; it adds K63-linked polyubiquitin chains onto stalled ribosomal proteins, especially those within the small subunit. Accordingly, its deletion alleviates stalling and increases the production of full-length-protein products from reporters engineered to stall^8,22–24^. Hel2 appears to recognize collided ribosomes^25,26^, which are expected to arise during stalling given that most mRNAs are occupied by multiple ribosomes such that collisions with lagging ribosomes are inevitable if downstream ribosomes stop translating. Subsequent ubiquitylation of stalled ribosomes provides the signal for the degradation of the aberrant mRNA, as either deletion of Hel2 or the introduction of mutations that prevent ribosomal protein ubiquitylation stabilizes the aberrant transcript^10,26,27^. In this capacity, Hel2 is considered to be a master regulator that connects NGD and RQC.

NGD and RQC have been studied almost exclusively in the context of artificial reporters that were genetically engineered to harbor roadblocks that stop ribosomes from translating. These blocks include: stable-RNA-secondary structures such as long stem loops, internal polyA sequences, stretches of rare codons, inhibitory codon pairs, and sequences encoding peptides that interact with the exit tunnel of the ribosome^1,2,23,28–30^. Although studies employing these reporters have been instrumental in unraveling the key mechanistic details related to NGD and RQC, the utility of these processes and their activation in response to different biological cues remain unclear.

We previously showed that mRNAs harboring 8-oxoguanosine (8-oxoG) stall translation in an in vitro reconstituted translation system, suggesting that oxidized mRNAs are targeted for NGD^31^. Furthermore, many alkylative adducts, especially those with a modified Watson–Crick face that are predicted to interrupt base-pairing during tRNA selection, also stall translation^32^. In addition, unwanted modification of nucleic acids, especially in the context of DNA damage, has been well documented to occur as a result of nucleobase reactivity with endogenous and exogenous agents^33,34^. One example is reactive oxygen species (ROS) that generate nucleobase adducts with altered base-pairing properties^35,36^. The adduct 8-oxoG is of particular note given its abundance, owing to the inherent reactivity of guanosine with ROS^37,38^, as well as its proclivity to adopt a *syn* conformation capable of Hoogsten-type basepair interactions with adenosine^36^. Conversely, alkylating agents can modify the nitrogen and oxygen atoms of the nucleobases^39^. Some of these modifications, including N1-methyladenosine (m1A), N1-methylguanosine (m1G), and N3-methylcytosine (m3C), are cytotoxic when present in DNA as they block replication and hence are predicted to stall translation^38,40^.

Although the above observations imply that cells have evolved pathways to recognize damaged mRNA and rapidly degrade it, the mechanistic details are not fully appreciated. Because transcripts that harbor damaged adducts are likely to stall translation, we hypothesize that damaged mRNA is subject to NGD while the encoded peptide is cleared by a RQC mechanism. Here, we tested this hypothesis and showed that both processes are activated by alkylation and oxidative stress to rapidly degrade chemically modified mRNAs and the resulting aberrant peptides encoded by them. Collectively, our data suggest that NGD and RQC evolved coordinately to cope with adduct-mediated ribosome stalling and highlight the potential burden of chemically modified mRNAs on cellular homeostasis.

## Results

### Xrn1 deletion leads to oxidized and alkylated adducts in mRNA

**Fig. 1:**
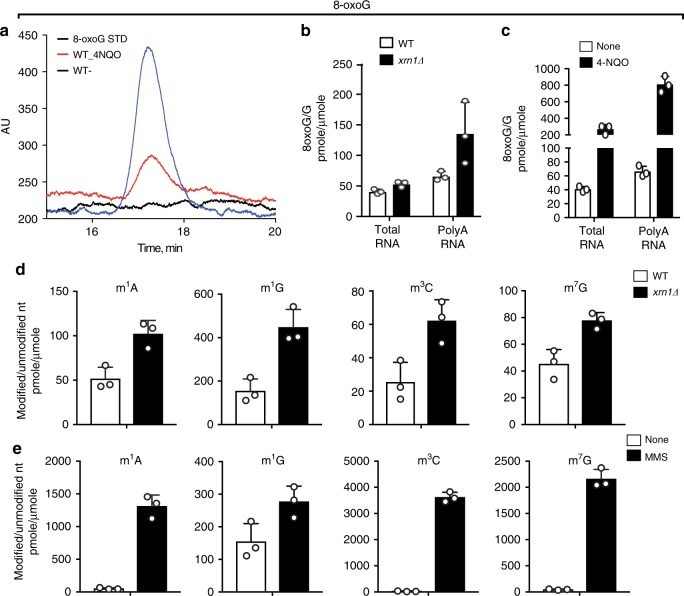
Oxidized and alkylated mRNAs accumulate in the absence of Xrn1. **a** A representative HPLC-ECD chromatogram used to quantify the concentration of 8-oxoG in our samples. **b** Bar graphs showing the ratio of 8-oxoG to G in total and polyA-RNA in presence and absence of *Xrn1*. **c** Bar graphs showing that the ratio of 8-oxoG to G in total and polyA-RNA significantly increases when 4-NQO is added to growing yeast cells. **d** Bar graphs demonstrating the ratio of the indicated alkylation adducts relative to their respective unmodified nucleoside in polyA-RNA in the presence and absence of Xrn1. **e** Same as **d** but in the absence and presence of MMS. In all cases, bars represent the average (±SD) of at least three independent biological replicates. Source data are provided as a Source Data file.

During NGD, regardless of the pathway involved, the aberrant mRNA is degraded by Xrn1^1,2^. Deletion of this factor is known to stabilize not only a full-length NGD reporter transcript but the cleaved 3′-fragment as well^2^. We reasoned that if damaged mRNA is subject to NGD, then deletion of *XRN1* should increase the levels of modified nucleotides in the mRNA pool such as the oxidation product 8-oxoG. We previously used competitive enzyme-linked immunosorbent assays (ELISAs) to show increased levels of 8-oxoG in *xrn1Δ* yeast cells^31^. As this method is not sufficiently sensitive or quantitative, we used here two more analytical methods, namely electrochemical detection (ECD) (Fig. 1a) coupled with HPLC^41^ and Liquid chromatography–mass spectrometry (LC-MS) to better quantify the adduct^42^. Total RNA was isolated from wild-type and mutant cells and subjected to two rounds of poly-dT purification to enrich for polyA-RNA (Fig. 1 and Supplementary Fig. 1a). Total RNA and mRNA-enriched samples were then treated with P1 nuclease and the resulting nucleotides were dephosphorylated to nucleosides using calf intestinal phosphatase (CIP) before analysis. Consistent with previous reports^43^, we measured under normal conditions a ratio of 8-oxoG to G of 4 × 10^−5^ and 3.7 × 10^−5^ in total RNA by ECD and LC-MS, respectively. As expected, this ratio did not change in *xrn1Δ* cells (Fig. 1b and Supplementary Fig. 1b, c). However, when polyA-RNA was specifically analyzed, the ratio of 8-oxoG to G increased by more than twofold when *XRN1* was deleted (Fig. 1b and Supplementary Fig. 1c), suggesting that oxidized mRNA is selectively prone to Xrn1-mediated quality control.

To add further support for our model that damaged mRNA in general is selectively targeted for degradation, we analyzed specific alkylative damage marks, including m1A, m1G, and m3C, which are known to disrupt base pairing^38,40^, as well as N7-methylguanosine (m7G), which does not disrupt base pairing. After processing of the RNA, the modified nucleosides were quantified by sensitive LC-MS methods^42^ that can detect 3.5–8.8 fmol of nucleoside (Supplementary Fig. 1e, f). Similar to our observations with 8-oxoG, deletion of *XRN1* significantly increased levels (two- to threefold) of the alkylative adducts known to disrupt Watson–Crick base pairing within mRNA (m1A, m1G, and m3C), whereas a more modest increase (~1.5 fold) was seen for m7G that does not modify Watson–Crick base pairing (Fig. 1d). As expected, deletion of *XRN1* has little to no effect on the levels of these modifications in total RNA given their natural presence in rRNA and tRNA (Supplementary Fig. 1g). Collectively, the data agreed with our model that modified mRNAs, for which base pairing with tRNAs could be significantly disrupted, are subject to quality control with Xrn1 playing a critical role in their prompt degradation.

### Oxidizing and alkylating agents increase modifications in mRNA

Our initial findings implicated Xrn1 in rapidly degrading chemically damaged mRNAs. We expect NGD to be responsible for this process whereby modified mRNAs are recognized by ribosomes, which in turn recruit downstream factors required for RNA degradation and RQC activation. To provide support for this model, we initially focused on increasing the levels of damaged mRNAs in yeast, which would allow us to assess whether different components of the quality-control pathways are activated. We took advantage of several damaging agents known to modify nucleic acids, including the UV-mimic 4-nitroquinoline 1-oxide (4-NQO), which generates ROS and hence the oxidation of G^44,45^, and methyl methanesulfonate (MMS), which alkylates RNA via S_N_2-reactive species that modify the exocyclic oxygen and nitrogen atoms within the nucleobase^46^. As expected, the addition of 4-NQO at 5 μg/mL for 30 min led to a > 10-fold increase of 8-oxoG in polyA-RNA, but no significant increase in total alkylated bases (Fig. 1a, c, Supplementary Fig. 1i). Similarly, the addition of MMS at 0.1% for 30 min produced a 30-fold, 160-fold and 50-fold increase of m1A, m3C, and m7G in polyA-RNA, respectively, but no significant changes to the level of total 8-oxoG (Fig. 1e, Supplementary Fig. 1d). We noted that the basal levels of these modified nucleosides in total RNA were much higher given their natural presence in rRNA and tRNA species^47^. As a result, the addition of MMS led to only a modest increase in their levels (Supplementary Fig. 1h). Regardless, our findings revealed that the addition of 4-NQO and MMS causes the widespread accumulation of damaged nucleotides in the mRNA, which in turn should pervasively stall ribosomes.

### 4-NQO and MMS increase ubiquitylation of nascent peptides

**Fig. 2:**
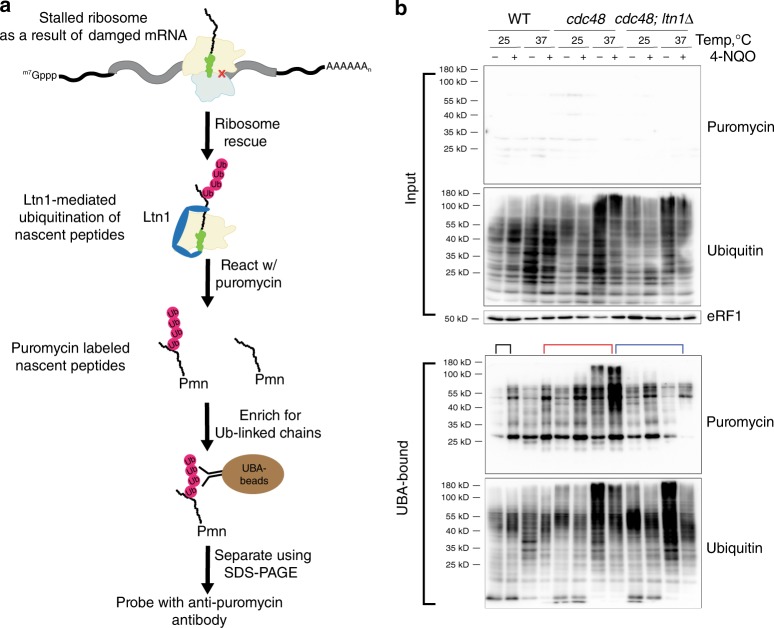
Ubiquitinated-nascent peptides accumulate in the presence of the oxidizing agent 4-NQO. **a** A schematic showing the experimental setup used to assess the amount of nascent peptides in the ubiquitinated pool. After the addition of 4-NQO, cells are harvested, lysed, and puromycin is added to react with stalled ribosomes. TUBE beads are then used to bind ubiquitinated proteins. The purified proteins are separated using SDS–PAGE and probed with anti-puromycin antibody. **b** Western blot analysis of the input and TUBE-bound samples in the absence and presence of 4-NQO for the wild-type, *cdc48-ts, ltn1Δ,* and *cdc48-ts;ltn1Δ* cells. Note the signal for puromycin in the input sample is faint relative to that for ubiquitin. Black lines compare the WT sample in the absence of the drug with that in its presence at the permissive temperature. Red lines compare wild-type cells with the cdc48-ts ones in the presence of the drug under the restrictive conditions. Blue lines compare the *cdc48-ts* to *cdc48-ts;ltn1Δ* cells at the restrictive temperature.

During RQC, the peptidyl-tRNA bound to the 60S subunit is recognized by Ltn1, which adds K48-linked polyubiquitin chains to the nascent peptide before its extraction by Cdc48 and presentation to the proteasome for degradation^9,16^. We reasoned that if 4-NQO increased the accumulation of 8-oxoG within mRNA, which in turn would stall ribosomes, a hyper-accumulation of ubiquitylated-nascent peptides upon addition of the chemical would be observed. The levels of these peptides should increase when Cdc48 is inactivated and decrease without Ltn1. As *CDC48* is an essential gene in yeast; we opted to use a temperature-sensitive allele for the analysis^16^. To specifically assess ubiquitylation of nascent peptides, we used puromycin, a ribosomal A-site mimic that reacts with peptidyl tRNAs on the ribosome and terminates protein synthesis, to label nascent peptides. Ubiquitylated forms of these peptides were then enriched for using Ubiquilin 1 Tandem UBA (TUBE2) beads and quantified by immunoblotting with anti-puromycin antibodies (Fig. 2a).

As expected, the addition of 4-NQO increased the amount of puromycylated peptides in the ubiquitylated pool regardless of the strain background (Fig. 2b). Although shifting wild-type cells from the permissive to the restrictive temperature had no observable effect on the accumulation of ubiquitylated and puromycylated nascent peptides after 4-NQO treatment, they were markedly increased at the restrictive temperature when *ts-cdc48* cells were used (Fig. 2b). This increase was also accompanied by an increase in the total levels of ubiquitylated peptides, as expected given the general role of Cdc48 in maintaining protein homeostasis^48^. By contrast, deletion of *LTN1* from these cells almost completely abolished the effects of 4-NQO on the accumulation of ubiquitylated-nascent peptides (Fig. 2b). This effect was not specific to oxidative damage, as the addition of MMS produces similar outcomes and leads to accumulation of puromycylated-ubiquitinated peptides (Supplementary Fig. 2). Taken together, these observations suggest that the addition of either 4-NQO or MMS stalls translation and activates RQC, which increases ubiquitylation of nascent peptides by Ltn1 via a route also involving CDC48.

### Alkylating and oxidizing agents lead to protein aggregation

Given that our data implicated Ltn1 in clearing incomplete-nascent peptides arising from chemically induced mRNA damage, we speculated that in *ltnΔ* cells, these peptides misfold and aggregate, which in turn would recruit chaperones. To visualize this potential aggregation, we exploited fusions of mCherry to the yeast-specific disaggregase HSP104^49^, and to the Ssa1 and Ssa3 chaperones, which are members of the HSP70 family, to look for chaperone-specific foci by fluorescence microscopy of wild-type and *ltn1Δ* cells grown under normal conditions or in the presence of oxidizing agents, alkylating agents, and translation inhibitors. Discernable foci of Ssa1- and Ssa3-mCherry were not observed under all conditions tested, suggesting that they do not help yeast cope with chemical-induced RNA damage (Supplementary Fig. 3a, b). More specifically, the lack of foci in *ltnΔ* backgrounds suggested that these factors play little to no role during ribosome stalling.

**Fig. 3:**
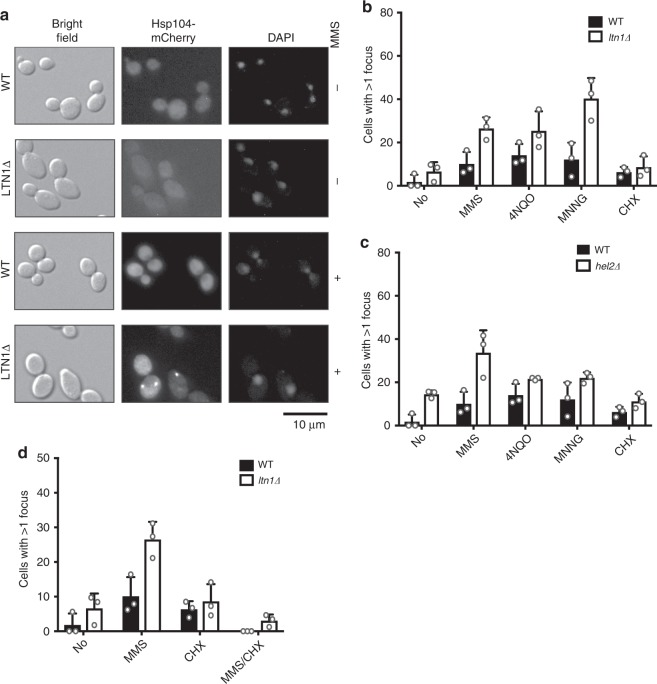
The addition of damaging agents leads to the accumulation of Hsp104-mCherry foci in the absence of Ltn1 and Hel2. **a** Representative images of the indicated cells in the absence and presence of MMS. Shown are brightfield, mCherry and DAPI signals. Scale bar is 10 μm. **b** Bar graph showing the quantification of cells with >1 focus in the presence of the specified drugs for WT and *ltn1Δ* cells. **c** Similar to **b**, using WT and *hel2Δ* cells. **d** Bar graph used to show the consequence of a cycloheximide pre-treatment on the effect of MMS on foci formation. In all cases, the average foci count of at least three biological repeats is plotted with the error bars representing the standard deviation around the mean. For each biological repeat, > 120 cells were counted. Source data are provided as a Source Data file.

Conversely, distinct foci decorated with Hsp104-mCherry were seen in >10% of wild-type cells when treated with the alkylating and oxidizing agents MMS, 4-NQO and MNNG, versus only 2% for untreated cells (Fig. 3a, b). Some of these Hsp104-dependent foci were likely generated by direct damage to the protein pool by these chemical agents. To resolve this possibility, we analyzed *ltnΔ* cells, which specifically acts on ribosomes to target nascent peptides during RQC, to sort out whether these foci arose by stalling-mediated production of incomplete peptides. Indeed, loss of Ltn1 increased the number of cells with Hsp104-mCherry foci from ~10% to 40–60% in the presence of the damaging agents (Fig. 3b). Hence, the effect of the chemical agents on the properties of mRNA was much more pronounced relative to their effect on protein, which in turn impacts its translation. In addition to Ltn1, the deletion of Hel2, which acts upstream and is required for RQC activation, led to a marked increase in the number of cells with Hsp104-mCherry foci in the presence of damaging agents (Fig. 3c).

Consistent with the idea that these aggregates contain nascent peptides evading RQC, inclusion of the translation inhibitor cycloheximide prior to MMS treatment caused the loss of these Hsp104-decorated foci (Fig. 3d). In particular, the number of *ltn1Δ* cells with foci decreased from ~25% to ~5%, which was close to those measured in the absence of any drugs, suggesting that active translation is required for forming Hsp104 foci. Notably, these foci appear distinct from P bodies^50^, as we saw no co-localization of Hsp104-mCherry with the P-body marker Dcp2-GFP (Supplementary Fig. 3c). Our data suggest that Hsp104 is recruited to aggregated nascent peptides generated during damaged mRNA-induced stalling when RQC is overwhelmed or incapable of ubiquitylating them for subsequent proteasome removal.

### Damaging agents trigger ribosomal protein ubiquitylation

**Fig. 4:**
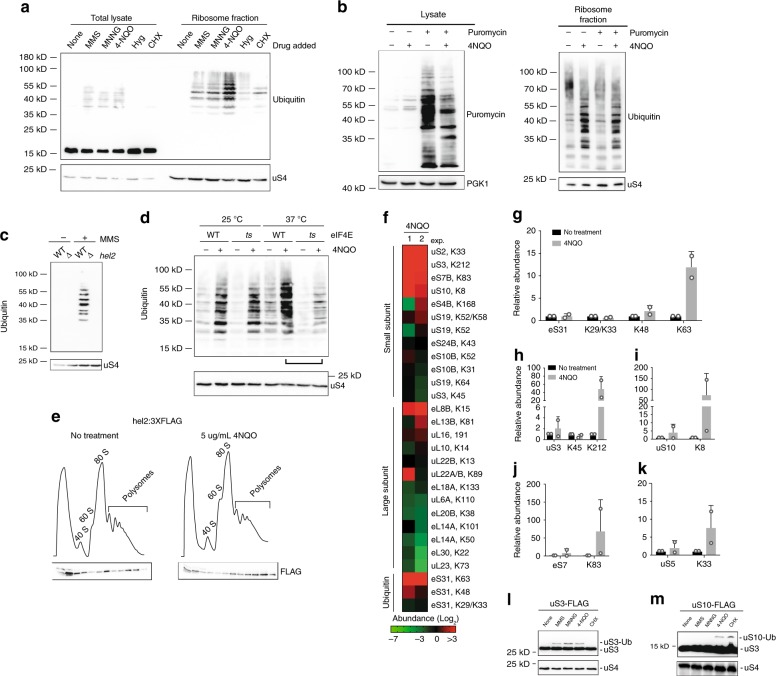
Ribosomal proteins are ubiquitinylated by Hel2 in the presence of damaging agents. **a** Western blot analysis of ubiquitinated proteins in total lysates and ribosome fractions in the presence of the indicated compounds. **b** Release of nascent peptides by puromycin does not affect the ubiquitination pattern of proteins in the ribosome fraction. Right panel is a western analysis of the puromycylation reactions under the indicated conditions. Left panel is a western analysis of ubiquitination of ribosome-enriched fraction in the presence and absence of puromycin upon the addition of 4-NQO. **c** Western-bot analysis showing that the ubiquitination of ribosomal proteins after MMS addition is completely inhibited when *HEL2* is deleted. **d** Western blot analysis used to assess the relative amount of ubiquitinated ribosomal proteins in the presence of 4-NQO when eIF4E, and hence initiation, is inactivated. Lines compare WT and *cdc33-ts* cells in the presence of 4-NQO at the restrictive temperature. **e** Sucrose-gradient fractionation of lysates obtained from endogenous *HEL2:* 3 × FLAG tag yeast cells in the absence and presence of 4-NQO. The traces follow the absorbance at 254 nm, below the traces is the western blot analysis of the fractions using anti-FLAG antibody. **f** Heat map showing scaled ubiquitination footprints of ribosome and ubiquitin-derived peptides in response to 4-NQO. The samples were digested with trypsin and enriched for diGly, prior to tandem MS analysis. Two independent biological replicates are shown and the modified lysine residues are indicated to the right of the ribosomal protein name. The peptides are organized based on their origin and subsequently ranked by their response to 4-NQO. **g** Bar graph showing the relative abundance of ubiquitin (eS31) and different ubiquitin chains. **h**–**k** Bar graphs showing the relative abundance of corresponding ribosomal proteins (on the left) and diGly modified peptides (on the right) in the absence and presence of 4-NQO (In all cases *n* = 2, ±SD). **l**–**m** Western blot analysis used to follow the ubiquitination of depicted ribosomal proteins in the presence of the indicated compounds. A C-terminal FLAG tag was added to the chromosomal copy of ribosomal protein genes. Source data are provided as a Source Data file.

As mentioned above, ribosomal protein ubiquitylation by Hel2 is a key signal for mediating the downstream events of NGD and RQC during stalling^21,25,26,51^. Hel2 recognizes an interface between the small subunits of collided ribosomes and adds K63-linked poly-ubiquitin chains to a number of core ribosomal subunits, including uS10, uS3, and eS7^21,25,26^. To address whether damaging agents also induce ribosomal protein ubiquitylation, we added MMS, MNNG, and 4-NQO to growing yeast cultures and assessed the level of protein ubiquitylation in total lysate and in purified ribosomes. Interestingly, although alkylating and oxidizing agents only subtly increased the overall level of ubiquitylation, they triggered robust ribosomal protein ubiquitylation as measured by immunoblotting with anti-ubiquitin antibodies (Fig. 4a). By contrast, the addition of the antibiotics cycloheximide and hygromycin, which both arrest translation at high concentrations and thus prevent ribosome collisions, triggered marginal ribosome ubiquitylation (Fig. 4a). To confirm the ubiquitylation pattern we observe in the presence of 4-NQO reflected ribosomal protein ubiquitylation and not that of nascent peptides, we treated the lysates with puromycin, which would release nascent peptides prior to ribosome enrichment. This puromycin pre-treatment had no discernable impact on the ubiquitylation of the ribosome-enriched samples (Fig. 4b). Therefore, the increased ubiquitylation upon exposure to oxidation and alkylation agents most likely reflected direct modification of ribosomal proteins.

We next sought to confirm that this pattern of ribosome ubiquitylation was distinct from those known to arise during oxidation and ER stress by assessing its dependence on E3s associated with RQC and non-functional ribosomal RNA decay (Fig. 4c, Supplementary Fig. 4a, b). Here, we analyzed the level of ribosomal ubiquitylation after treatment with MMS in the presence and absence of either Hel2, Mms21, or Rtt101, the latter two of which are known to function during non-functional ribosome decay^52,53^. A complete loss of ribosomal ubiquitylation was only observed in *hel2Δ* cells (Fig. 4c, Supplementary Fig. 4a, b), supporting the idea that damaging agents-induced ubiquitylation depends on translation of the aberrant mRNA. Complementing these cells with a plasmid-borne *HEL2* restored ubiquitylation to normal levels, whereas an empty vector did not (Supplementary Fig. 4c). Using an antibody specific for ubiquitin internally linked through K63, we confirmed that the ubiquitin chains bound to ribosomal proteins were mostly connected via this lysine (Supplementary Fig. 4c), in agreement with the specificity of Hel2 for K63 polyubiquitylation.

We next showed that ribosomal protein ubiquitylation depends on active translation using two different approaches. For the first approach, we exploited an eIF4E temperature-sensitive allele (*cdc33-ts4–2*) to suppress translation initiation. Whereas shifting wild-type cells to the restrictive temperature had little effect on 4-NQO-induced ribosomal protein ubiquitylation, shifting the *cdc33-ts4–2* cells to the restrictive temperature greatly reduced this modification (Fig. 4d). For the second approach, we used abrupt glucose deprivation to induce ribosome runoff and examined how this starvation affected ribosomal protein ubiquitylation. As shown in Supplementary Fig. 4d, ribosome runoff prior to 4-NQO addition almost completely inhibited ribosomal proteins ubiquitylation, implying that ribosomes must be actively translating for nucleobase-damaging agents to trigger their ubiquitylation.

### Hel2 associates with polysomes in the presence of stress

To demonstrate that Hel2 is recruited to ribosomes upon the addition of oxidizing and alkylating agents, we investigated the association of this E3 with polysomes under different conditions. To facilitate detection by immunoblot assays, the endogenous *HEL2* gene was tagged with a C-terminal FLAG epitope. The samples were cross-linked with formaldehyde to stabilize any transient interactions prior to sucrose-gradient fractionation of polysomes. As expected, Hel2-FLAG was predominantly found with the light fractions and appears not to be recruited efficiently to the ribosomes. In the presence of 4-NQO and MMS, Hel2 predominantly associated with the polysome fraction (Fig. 4e). As Hel2 is documented to recognize collided ribosomes, these observations suggest that alkylating and oxidizing agents generate ribosome collisions, which together with the accumulation of ubiquitylated ribosomal proteins (Fig. 4a, b), implies that alkylating and oxidizing agents activate Hel2 through ribosome stalling.

### Characterization of ubiquitin-modified-ribosomal proteins

To interrogate the dynamics of ribosome ubiquitylation in response to RNA-damaging agents, we use a quantitative-proteomic approach to measure ubiquitylation that combined diglycine (diGly)-modified immuno-affinity enrichment followed by tandem MS to follow site-specific ubiquitin additions onto individual ribosome proteins^54^. MS/MS analysis of individual peptides from the diGly-peptide-enriched pool revealed that the ubiquitylation status of individual proteins within both the large (60S) and small (40S) subunits was altered upon 4-NQO treatment (Fig. 4f). The majority of proteins displaying increased ubiquitylation in the presence of 4-NQO were located on the 40S subunit (Fig. 4f), whereas the majority of proteins displaying a decreased ubiquitylation in the presence of 4-NQO were located on the 60S subunit (Fig. 4f) (Supplementary Table 1).

Given that the nature of the internal linkage used for polyubiquitylation can have a marked effect on downstream events^55^, we further characterized by MS/MS the dynamics of polyubiquitylation linkages during the 4-NQO treatment. Interestingly, whereas the total amount of ubiquitin bound to purified ribosomes remained unchanged after 4-NQO treatment, the peptide derived from ubiquitins internally linked via K63 increased dramatically (>10-fold) (Fig. 4g). By contrast, the peptide derived from ubiquitins internally linked via K48 increased only moderately (1–3-fold) in the presence of the compound (Fig. 4g). Finally, we identified several lysine residues on the ribosomal proteins that are highly ubiquitylated as a result of 4-NQO treatment. These proteins include: K212 on uS3, K8 on uS10, K83 on eS7B, and K33 on uS5 (Fig. 4h–k). We further validated the ubiquitylation events on uS3, uS10, eS7, and uS5 by immunoblotting (Fig. 4l, m, Supplementary Fig. 4f, g).

### DNA damage does not elicit ribosomal protein ubiquitylation

**Fig. 5:**
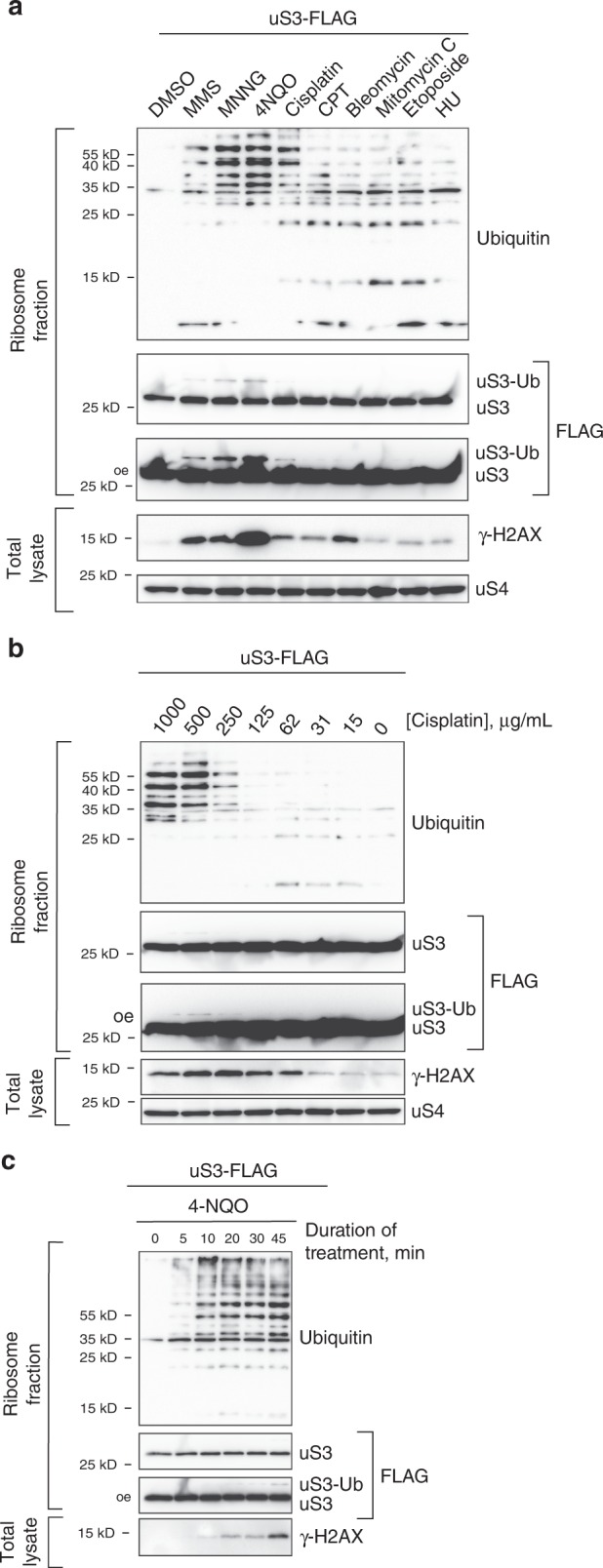
Agents that strictly compromise the integrity of DNA do not lead to ribosomal proteins ubiquitination. **a** Western blot analysis of ribosomal protein ubiquitination and uS3 in the presence of the indicated compounds. Phosphorylation of H2AX was used to assess the activation of the DNA-damage response. **b** Western blot analysis of ribosomal proteins ubiquitination as a function of increasing cisplatin concentrations. **c** Western blot analysis of ribosomal protein ubiquitination and H2AX phosphorylation as a function of time after the addition 4-NQO. Note ribosomal protein ubiquitination is observed only after 5 min of incubation and saturates at the ten-minute mark. In contrast, γ-H2AX signal appears not to near saturation after 45 min of incubation. Source data are provided as a Source Data file.

To provide further support for our model that MMS, MNNG, and 4-NQO activate RQC through their modification of RNA and not DNA, we examined the effect of agents that specifically target DNA on ribosomal protein ubiquitylation. In particular we tested: cisplatin (an inter-strand crosslinker), camptothecin (a topoisomerase I inhibitor), bleomycin (induces DNA-strand breaks), mitomycin C (DNA crosslinker), etoposide (a topoisomerase II inhibitor) and hydroxyurea (depletes the dNTP pool)^56^. As expected, the addition of all of these agents did little to increase the ubiquitylation state of ribosomal proteins and uS3. At the same time, they all elicited a DNA-damage response to various extents as assessed by phosphorylation of H2AX (accumulation of γH2AX) (Fig. 5a). This is similar to what has been observed in mammalian cells, for which the additions of etoposide, mitomycin C, and cisplatin were not found to impact ribosome ubiquitylation^57^.

Interestingly, the only drug that induced appreciable ubiquitylation of ribosomal proteins—at least as compared with MMS, MNNG, and 4-NQO—was cisplatin, which had been documented to cross-link ribosomes^58^, suggesting that it also affects translation and activates RQC (Fig. 5a). Indeed, addition of cisplatin at high concentrations was accompanied by moderately increased ribosomal protein ubiquitylation (Fig. 5b). Thus, we concluded that activation of RQC by damaging agents results from their impact on RNA properties and not their influence on DNA metabolism.

To assess the cellular response to the presence of damaging agents through ribosomal protein ubiquitylation, we examined these modifications as a function of time upon the addition of 4-NQO. Interestingly, we detected the accumulation of ubiquitylated ribosomal proteins within 5 min of treatment with the response saturated by 10-min. In contrast, the accumulation of γH2AX was not saturated after 45 min of incubation (Fig. 5c). These observations indicate that RNA is modified faster than DNA, with subsequent ribosome stalling and activation of RQC occurring shortly thereafter.

### Yeast lacking NGD and RQC components are sensitive to damage

**Fig. 6:**
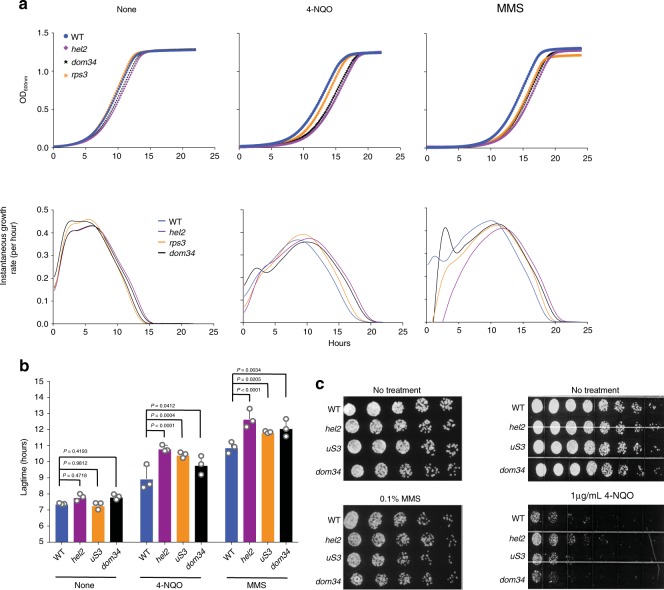
Mutations in NGD and RQC components render cells sensitive to 4-NQO and MMS addition. **a** Top is a plot of OD_600nm_ over time of the indicated strains after a mock treatment or a 30-minute challenge with 4-NQO (1 μg/mL) or MMS (0.1% MMS). Bottom is a plot of the first derivative of the data on top to calculate the instantaneous growth rate and the corresponding lag time needed to reach the maximum rate. Data were collected in technical duplicates from three biological replicates. **b** A bar graph showing the determined lag time from **a**. The mean of three biological replicates is plotted and the error bars represent the standard deviation. *p* value from two-way ANOVA test is indicated. Source data are provided as a Source Data file. **c** Images of spot assays performed using the indicated strains, after mock treatment or a 30-minute challenge using 4-NQO (1 μg/mL) or MMS (0.1% MMS).

Thus far, our analyses have focused on the details of RQC activation in response to damaging agents through their impact on RNA, but whether the inability to do so diminishes cellular fitness remained unclear. To examine whether failure to activate RQC renders cells sensitive to oxidizing and alkylating agents, we tested if yeast strains harboring mutations in various components of NGD and RQC would recover poorly from oxidation and alkylation stress. In brief, cells were treated with 1 μg/mL 4-NQO and 0.1% MMS for 30 min, washed with prewarmed fresh medium, and then monitored continuously for growth (Fig. 6a). In the absence of any treatment, we observed little to no difference in lag time or growth rate among the strains, as assessed by the first derivative of growth curves (Fig. 6b). Upon a 4-NQO challenge, *hel2Δ*; *rps3-R116, R117A*; *dom34Δ*; and *ski2Δ* strains had significantly increased lag times relative to the wild-type cells (Fig. 6b), whereas the lag time was unchanged in the *hsp104Δ* strain (Supplementary Fig. 5). Interestingly, *ltn1Δ* cells actually displayed a faster recovery as revealed by its lag time of 7.5 hr relative to 9 hr for wild type, but the additional deletion of *HSP104* from these cells significantly increased the recovery time (Fig. 6b, Supplementary Fig. 5). These observations suggest that in the absence of Ltn1, Hsp104 clears misfolded nascent peptides that fail to be ubiquitylated by this E3. Deletion of both factors then renders the cells sensitive to nascent peptides that accumulate from stalled translation of damaged mRNA. We note that treatment of NGD- and RQC-defective cells with the drugs for 30 min showed no differences in cellular death relative to wild-type as assessed by spot assays (Fig. 6c), suggesting that the defects we saw in growth rate were indeed caused by increased lag time post treatment. Collectively, our data imply that NGD and RQC play an important role in coping with chemical insults that alter the chemical properties of RNA.

### One 8-oxoG adduct in the coding sequence destabilizes mRNAs

**Fig. 7:**
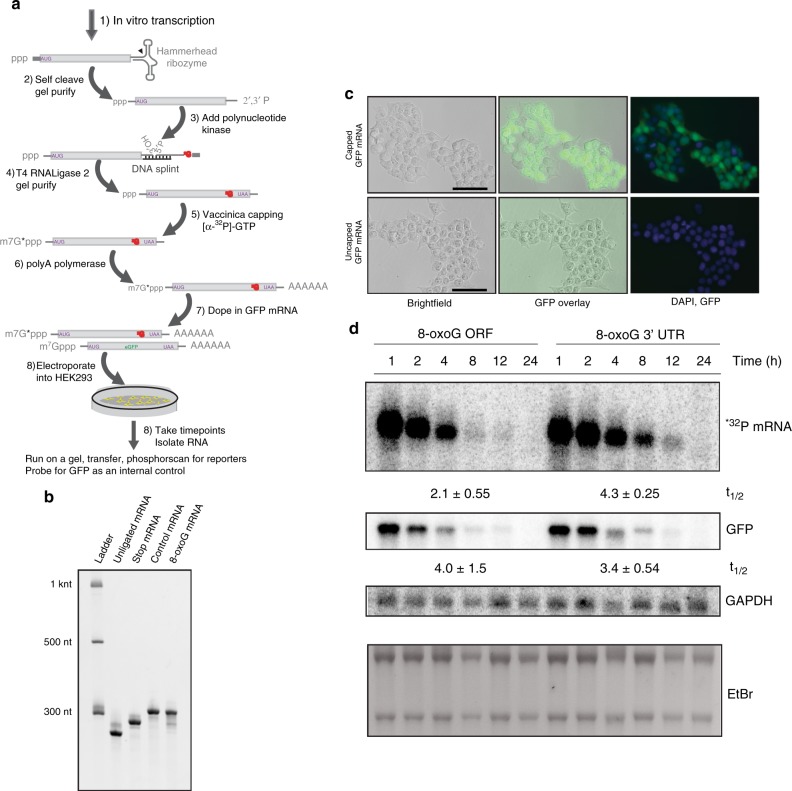
The presence of one 8-oxoG adduct in the ORF of an mRNA leads to accelerated rate of decay in HEK293 cells. **a** Schematic showing the steps used to make mRNA reporters with 8-oxoG in the ORF. **b** Ethidium-bromide-stained PAGE gel used to follow the construction of the mRNA showing that they migrate as would be predicted based on their size. **c** Microscope images of HEK293 cells electroporated with capped and uncapped eGFP mRNA used to assess electroporation efficiency. Shown are the brightfield, and GFP- and DAPI-fluorescence signals. Scale bar is 50 μm. **d** Northern analysis to measure the decay of the indicated reporters and the control GFP mRNAs post electroporation. Reporters were visualized directly by exposing the nylon transfer to a phosphorimager screen. GFP and GAPDH were visualized using a radiolabeled DNA probe. At the bottom is the ethidium-bromide stained agarose gel. The half-lives were determined from duplicates (±SD).

Until now, our analysis has focused on the effect of mRNA alkylation and oxidation on the activation of NGD and RQC using compounds that target RNA indiscriminately, which likely impacts bases throughout the RNA strand, resulting in the formation of a plethora of adducts in various locations on the RNA^31,59^. As a result, we sought to examine how the presence of one disruptive adduct might influence turnover of an mRNA. We opted to use HEK293 cells instead of yeast, because that allowed us to use electroporation to efficiently deliver in vitro-generated mRNAs to these cells. In brief, a short RNA containing an 8-oxoG was chemically synthesized and ligated to an in vitro-transcribed mRNA, whose end was healed with a hammerhead ribozyme (Fig. 7a, b). The resulting RNA was polyadenylated using polyA polymerase and capped with vaccinia capping enzyme in the presence of [α-^32^P]-GTP, which allowed us to monitor the fate of capped mRNAs specifically. Two constructs were generated with only one difference: one that harbored 8-oxoG in the ORF and another that positioned the adduct in the 3′-UTR (Supplementary Table 2). The former was expected to be subject to NGD, whereas the latter was not. As a control, we also co-electroporated a capped and polyadenylated eGFP mRNA, which allowed us to measure electroporation efficiency, found to be >90% (calculated between 92 and 96% across three experiments) (Fig. 7c).

To confirm that in vitro-generated mRNAs, like those synthesized in vivo are efficiently targeted by mRNA-surveillance pathways, we also engineered a control construct without a translation stop codon. This “no-stop” reporter also contained a polyA tail whose translation by the ribosome should target the RNA for non-stop decay (NSD). Indeed, when these mRNA reporters were electroporated into HEK293 cells, the control mRNA decayed with a half-life of ~5.3 hr, whereas the non-stop NSD version turned over rapidly with a half-life of <1 hr (Supplementary Fig. 6a). Having established that RQC processes appear capable of targeting mRNAs synthesized in vitro, we electroporated the 8-oxoG-ORF- or UTR-containing mRNAs into HEK293 cells and followed their turnover (Fig. 7d). In agreement with our hypothesis that mRNAs containing 8-oxoG in the coding region are subject to NGD, we measured a half-life of ~2.1 hr for the ORF-modified transcript versus ~4.3 hr for the UTR-modified one (Fig. 7d). Similarly, a control-stop mRNA reporter, which replaced the 8-oxoG where the ribosome is expected to stall with a stop codon, decayed with a half-life of 7 hr (Supplementary Fig. 6b). We note that these differences in half-lives are similar to what others have reported for NGD reporters with stalling sequences^1,60^. Furthermore, the undistinguished half-life for the eGFP mRNA of ~3.5–4 hr among the experiments confirmed that the differences we observe in the half-lives between the two reporters was caused by inherent differences between the two mRNAs and no other experimental factors. Taken together, our results strongly indicate that translation and hence the ribosome itself has an important role in governing the stability of adduct-containing mRNAs.

## Discussion

All organisms rely on quality-control mechanisms to deal with defective biological molecules. In addition to inherent mistakes that occur during the generation of these molecules, chemical agents can alter them following synthesis^61,62^. Nucleic acids are especially vulnerable to such insults given the inherent reactivity of the oxygen and nitrogen atoms of the nucleobases^63^. Two classes of endogenous and exogenous damaging agents that are particularly deleterious include ROS and alkylating agents^33,35,61^. Endogenous ROS arise as byproducts of mitochondrial respiration and their levels are significantly influenced by cellular metabolism^64^. For instance, tissues with high-energy demands such as neurons accumulate higher levels of ROS^65,66^. In addition to endogenous ROS, cells become exposed to exogenous agents that can increase ROS levels, include ionizing radiation, ultraviolet light, pollutants, and many toxins^63^. Similarly, alkylating agents appear endogenously, with the universal-methylating agent *S*-adenosyl methionine (SAM) being one of the most abundant^67^. SAM reacts with nucleic acids in vitro to form various modified bases^68^. As examples of exogenous alkylating agents, chemotherapeutics such as cyclophosphamide, streptozotocin, and temozolomide are known to target RNA in addition to DNA^69^. It should come as no surprise then that oxidized and alkylated mRNA molecules accumulate in vivo^70,71^, but whether cells are impacted is not immediately evident.

Given the transient nature of mRNA, it was once assumed that damage is of little consequence. However, the discovery that cells evolved multiple pathways to cope with defective mRNAs has significantly altered this view^38,63^. Furthermore, we have also known for some time that the abundance of oxidized RNA is correlated with various neurodegenerative diseases^72^, suggesting that cells have evolved pathways to deal with chemically damaged mRNA. These ideas are bolstered by findings that the genomes of almost all organisms and some viruses encode dealkylases involved in RNA repair^73^.

First, hints about quality-control pathways capable of degrading chemically damaged mRNAs came from studies showing that oxidized mRNAs turnover faster relative to undamaged versons^74^. The mechanism(s) by which these mRNAs are selectively recognized were not immediately obvious^31^; mainly because the effects of the modified bases on mRNA function was unknown. In the past few years, we and a number of other groups have begun to dissect the effects of modified bases on protein synthesis using in vitro approaches^31,32,75^. Interestingly, although some of the adducts behave as expected based on their structure and the equivalent studies on DNA replication (for example, m1A severely inhibits protein synthesis^32^), others have revealed unexpected effects on ribosomes. Of particular note is 8-oxoG, which mispairs with A during DNA replication by adopting the *syn* conformation, but on ribosomes severely inhibits protein synthesis, presumably because of its failure to adopt the *syn* conformation in the decoding center^31^. These findings suggested that chemically damaged mRNA triggers NGD and the associated RQC. In agreement with this scenario, we recently showed that 8-oxoG mRNAs accumulate in the absence of *XRN1* in yeast and associate with polyribosomes^31^. These findings suggest that oxidized mRNAs are subject to NGD, and subsequent degradation of the 3′-fragment by Xrn1^1,2^. Here, we expanded on these observations and provide evidence that other chemically modified bases including m1A, m3C and m1G accumulate in mRNA, when *XRN1* is deleted (Fig. 1d). As alluded to earlier, all of these adducts are either known or predicted to stall protein synthesis by severely disrupting Watson–Crick base pairing. Although these findings do not directly demonstrate that damaged mRNA is subject to NGD, they are consistent with the notion.

Typically, studies on damaged RNA have largely relied upon treating cells or bulk RNA with chemically modifying agents to assess their effect. To take a more precise look at how oxidizing agents impact mRNAs and their translation, we undertook a novel approach, using site-specifically modified mRNAs to assess whether mammalian cells recognize and remove these transcripts from the translating pool. By introducing a single 8-oxo-G modification to either the ORF or the 3′-UTR, we could measure significant differences in mRNA half-life; the mRNA harboring the modification in its ORF was degraded twice as fast as one with the modification in its UTR (Fig. 7). This faster degradation implied that cells not only recognize damaged mRNA, but exploit the translating ribosome to identify it and target it for breakdown, likely through NGD.

To provide further support for our model that NGD and RQC help cells cope with damaged mRNAs and the derived defective protein products, we took advantage of chemicals capable of modifying nucleic acids. In particular, we used both the alkylating agents MMS and MNNG, and the oxidizing agent 4-NQO. Indeed, we showed using LC-MS/MS that the addition of MMS to yeast induced an order of magnitude increase in the alkylative adducts m1A and m3C in mRNA (Fig. 1e), whereas the addition of 4-NQO led to a marked increase in RNAs harboring 8-oxoG (Fig. 1c). Given the strong increase in these damage marks, we predicted that accumulation of the damaged transcripts would induce widespread ribosome stalling. By examining labeled ubiquitylated-nascent peptides, we provided compelling evidence indicating that alkylative and oxidative stress activates RQC. First, the addition of MMS and 4-NQO was accompanied by an increase in ubiquitylated puromycylated peptides (Fig. 2b, Supplementary Fig. 2), suggestive of an increase in ribosome-associated quality control of nascent peptides. Interestingly, the addition of damaging agents had little to no effect on the total levels of ubiquitylated peptides (Fig. 2b), suggesting that the drugs do not significantly affect full-length proteins, at least at the drug concentrations used. Second, inactivation of Cdc48 via the *ts-cdc48* allele induced a marked increase in ubiquitylated puromycylated peptides (Fig. 2b). Cdc48 functions within the ubiquitin system, where it extracts defective proteins from complexes and delivers them to the proteasome^9^. In addition to its involvement in RQC, the CDC48 has role(s) in other quality control processes, including ERAD and autophagy^76–79^. Notably, our observations that Cdc48 inactivation in the presence of MMS, MNNG, or 4-NQO leads to increased levels of ubiquitylated peptides that are also puromycylated (Fig. 2b) suggests that the compounds appreciably alter mRNA translation. Third, deletion of *LTN1*, the E3 responsible for ubiquitylating nascent peptides during RQC, suppresses the effects of *cdc48-ts* in the presence of alkylating and oxidizing stress and greatly reduced the amount of puromycylated peptides to normal levels (Fig. 2b, Supplementary Fig. 2). Collectively, these findings suggest that not only is mRNA exceptionally susceptible to chemical insults, but also that resulting chemical modifications significantly impact its translation.

Although the above observations highlight the potential deleterious effect of chemical modification to the function of mRNA, they do not necessarily show that this alteration is sufficiently adverse to influence cellular fitness. However, our findings that cells lacking Ltn1 accumulate protein aggregates in the presence of chemical agents, as visualized through Hsp104 foci (Fig. 3), shows that mRNA damage could indeed reduce cellular proteostasis. We are not the first group to report that Hsp104-containg foci coalesce in the presence of damaging agents^80–82^, however this accretion was attributed to damage to DNA and not to RNA. Furthermore, the relationship between these foci, chemical damage, and RQC has not been explored previously. Thus, we identified an unappreciated role for Hsp104 in stalling-associated-protein defects as a potential failsafe for RQC. In agreement with this model, removal of LTN1 or HSP104 singly did not make yeast sensitive to damaging agents, but the combined elimination of both factors rendered the cells sensitive (Supplementary Fig. 5).

It has been well established that ribosomal ubiquitylation is required for ribosome RQC and ribosome rescue at translational stalls in both yeast and mammalian cells^20,21,25,26^. Our observations that damaging agents induce ubiquitylation of ribosomes supports our hypothesis that these compounds cause translational stresses through stalling (Figs. 3, 4). Recent cryoelectron microscopy studies provided convincing evidence that the two 40S subunits on collided ribosomes form a unique interface that discriminates stalled from active ribosomes and provides a potential binding site for Hel2^25,26^. Given its ability to stall ribosomes, it is not surprising that 40S subunit ubiquitylation is particularly robust after 4-NQO treatment (Fig. 4f). Previous proteomics studies identified ubiquitylation events on specific lysine residues within stalled yeast ribosomes during RQC induction, including lysines on uS3 (K212), uS10 (K8), and eS7A/B^21,83^. We hypothesize that damaging agents such as 4-NQO cause the ribosome to stall and subsequently elicits a similar ribosome ubiquitylation pattern. In agreement, our MS analyses show that all of the lysines that are ubiquitylated upon stalling are similarly targeted in the presence of 4-NQO (Fig. 4f). For example, the previously identified stalling target on uS5 (K33) is highly ubiquitylated in the presence of 4-NQO (Fig. 4k, Supplementary Fig. 4g). In agreement, uS5 is located on the interface between two collided 40S subunits close to uS3 in the entry tunnel, and was identified as a possible regulatory response to the accumulation of unfolded proteins in yeast^57^.

Although both K48- and K63-linked polyubiquitylation on ribosomes have been reported during RQC^21^, Hel2-mediated K63-linked polyubiquitination is required for both NGD-induced mRNA degradation, and subunit dissociation and 18S non-functional rRNA decay^26,83^. The observation that Hel2-dependent-K63-linked ubiquitylation is highly enriched upon 4-NQO treatment reinforces the critical role of the RQC pathway in coping with RNA damage (Fig. 4g). K63-linked polyubiquitin chains play an essential role in signal amplification processes for various cellular stress responses, including DNA repair and nuclear factor κB activation^84^. Its role in signaling has recently expanded to translation as it was reported that K63-linked ubiquitylation of ribosomal proteins activates multiple downstream quality control pathways, and the modification by Hel2 provides a platform for the interaction of ribosomes with other E3s or regulatory components^21,83,85^. Combined with the prevalence of damaging adducts in mRNA in the absence of *Xrn1* (Fig. 1d), the activation of Hel2-mediated ribosomal ubiquitylation might have evolved to resolve the translational stresses induced by mRNA damage.

Finally, the observation that ribosomal proteins ubiquitylation appears faster than H2A.X phosphorylation^86^ offers two notions: (1) RNA damage occurs on a much faster time scale relative to DNA damage, (2) stalling of translation appears sooner than changes to DNA metabolism (for instance, changes to replication). The former implication is reinforced by the chemical nature of RNA relative to DNA; RNA, and specifically mRNA, are relatively protein-free and single-stranded, thus rendering the nitrogen and oxygen atoms of the Watson–Crick face of the nucleobase vulnerable to chemical attack^38^. The latter implication suggests a potential avenue for how cells respond to chemical insults; that organisms, in principle, utilize ribosome stalling and the associated ribosomal ubiquitylation to mount a protective response before DNA becomes damaged.

## Methods

### Yeast strains and plasmid constructs

Yeast strains used in this study are listed in Supplementary Table 3. Unless otherwise stated, yeast cells were grown at 30 °C in YPD media. Deletion, mutant and chromosomally tagged yeast strains were constructed in the BY4741 background (*MATa; his3Δ1; leu2Δ0; met15Δ0; ura3Δ0*) with PCR-based disruption techniques. Plasmids used are listed in Supplementary Table 4. Unless otherwise stated, MMS, MNNG, 4-NQO, cycloheximide and hygromycin were added to final concentrations of 0.1%, 20 μg/mL, 5 μg/mL, 100 μg/mL, and 200 μg/mL, respectively. DNA-damaging agents of cisplatin, camptothecin, bleomycin, mitomycin C, etoposide, and hydroxyurea were added to final concentrations of 250 μg/mL, 30 μg/mL, 60 μg/mL, 2.6 mM, 1 mg/mL, and 100 mM, respectively. Cells were incubated with drug for 30 min, unless stated otherwise. Oligonucleotide sequences are listed in Supplementary Table 5.

### Quantification of RNA modifications by LC-MS/MS

Yeast cells were grown overnight in YPD. The cultures were then diluted to OD 0.05 in the same media and grown to OD 0.5–0.8. When indicated, cultures were treated with damaging agents for 30 min. RNA was isolated using the hot-phenol extraction method^51^. Two rounds of polydT pulldown were used to enrich mRNAs. Total RNA (1 µg) and Poly(A) mRNA (0.1 µg) were digested by nuclease P1 (10 Units) at 50 °C for 14–16 hr. Following the addition of Tris pH 7.5 to a final concentration of 100 mM to adjust the pH, calf intestinal alkaline phosphatase (CIP, NEB) was added (2–2.5 Units) and the reaction was incubated at 37 °C for an additional 1 hr to convert nucleotide 5′-monophosophates to their respective nucleosides. The RNA samples were diluted to 30–50 μL and filtered (0.22 μm pore size). In all, 10–15 μL of the sample was used for LC-MS/MS. In brief, nucleosides were separated on a C18 column (Zorbax Eclipse Plus C18 column, 2.1 × 50 mm, 1.8 Micron) paired with an Agilent 6490 QQQ triple-quadrupole LC mass spectrometer using multiple-reaction monitoring in positive-ion mode. The nucleosides were quantified using the retention time of the pure standards and the nucleoside to base ion mass transitions of 268.1–136 (A), 244.1–112 (C), 284.2–152 (G), 245.1–113 (U), 300–168.1 (8-oxoG), 282.2–150 (m1A), 298–166 (m1G), 258–126 (m3C), 282.1–150 (m6A), 298–166 (m7G). Standard-calibration curves were generated for each nucleoside by fitting the signal intensities against concentrations of pure-nucleoside preparations. The curves were used to determine the concentration of the respective nucleoside in the sample. The A, G, and C standards were purchased from ACROS ORGANICS; U was purchased from TCI America. m7G, m1G, and m3C were purchased from Carbosynth, m6G, and m6A were purchased from Berry’s Associates, and m1A was from Cayman Chemical Company. The unmodified nucleosides were quantified using the UV-Vis absorbance data that were recorded by a diode-array detector The modification level on the nucleosides was calculated as the ratio of modified to unmodified. Data were analyzed using Excel (Microsoft) and Prism 7 (Graphpad) software.

### Polysome analysis

Yeast cells were grown to mid-log phase before cycloheximide (CHX) was added to the indicated concentration. The cultures were chilled on ice and centrifuged at 4 °C. Cells were then resuspended in polysome-lysis buffer (20 mM Tris pH 7.5, 140 mM KCl, 5 mM MgCl_2_, 0.5 mM DTT, 0.1% Triton-100, CHX to the indicated concentration, 200 μg/mL heparin), washed once and lysed with glass beads using a FastPrep-24 (MP Biomedical). Supernatants from cleared lysates corresponding to 0.5–1 mg of total RNA were layered over a 10–50% sucrose gradient and centrifuged at 35,000 rpm for 160 min in an SW41Ti (Beckman) swinging bucket rotor. Gradients were then fractionated using a Brandel tube-piercing system combined with continuous absorbance reading at 254 nm.

### Western blot analysis

Cells were grown to mid-log-phase and treated with chemicals. The total lysates were harvested with ice-cold lysis buffer (150 μl of 1.85 M NaOH, 7.5% beta-mercaptoethanol) and 55% trichloroacetic acid. Lysates were then pelleted and resuspended in an appropriate volume of HU buffer (normalized to the number of cells harvested). HU buffer is composed of 8 M urea, 5% sodium dodecyl sulphate (SDS), 200 mM Tris pH 6.8, 1 mM ethylenediaminetetraacetic acid (EDTA), bromophenol blue, 100 mM DTT. Proteins were separated by SDS–PAGE and analyzed by immunoblotting. The following antibodies were used: mouse anti-ubiquitin HRP (Santa Cruz, Cat#: sc8017; 1:2,000 v/v dilution), mouse anti-FLAG (Sigma; Cat#: F1804; 1:5,000 v/v dilution), mouse anti-puromycin (Millipore, Cat#: MABE343; 1:5,000 v/v dilution), rabbit anti-uS4 (Rps9) (Abcam, Cat#:ab117861; 1:5,000 v/v dilution), rabbit anti-phospho-Histone H2A.X (Cell Signaling, Cat#: ab11174; 1:3,000 v/v dilution), anti-ubiquitin (K48-linked-ubiquitin chains) (Cell Signaling, Cat#: 4289 s; 1:3,000 v/v dilution, linkage-specific k48), anti-ubiquitin (K63-linked-ubiquitin chains) (Cell Signaling, Cat#: ab179434; 1:3,000 v/v dilution, linkage-specific k63), and rabbit anti-eRF1^87^ (1:2000 dilution). Secondary antibodies of goat anti mouse IgG (Thermo Scientific, Cat#: 31430) and goat anti rabbit IgG (Thermo Scientific, Cat#: 31460) were used at (1:10,000 v/v dilution).

To examine the ubiquitination on ribosomes, yeast cells were first harvested and washed with ice-cold polysome-wash buffer (20 mM Tris pH 7.5, 140 mM KCl, 5 mM MgCl2, 5 mM DTT). Cell pellets were then resuspended in 200–500 µL of polysome-lysis buffer (100 µg/mL Heparin, 1 mM pheylmethylsulfonyl fluoride (PMSF), 20 mM Tris pH 7.5, 140 mM KCl, 5 mM MgCl2, 5 mM *N*-ethylmaleimide (NEM), 5 mM DTT]. Cells were homogenized by FastPrep-24 (MP Biomedical #116004500) with 200–500 µL of glass beads (Sigma, acid-free, Ø 425–600 µm, #G8772–500G) at 4 °C. Cells were processed at top speed for 1 min with 1 min resting on ice for five cycles. Cell debris was removed by pelleting at 15,000 × *g* for 10 min at 4 °C. Ribosome-enriched fractions were pelleted over a 0.5 mL sucrose cushion (1.1 M sucrose, 20 mM Tris-HCl pH 7.5, 500 mM NH_4_Cl, 0.5 mM EDTA, 10 mM MgCl_2_, 5 mM NEM, 1 mM PMSF) by centrifugation at 287,000 × *g* for 2 hr at 4 °C in an MLA rotor. Ribosomes were resuspended in 15–30 μl of RNase-free water. The concentration of ribosomes was measured using absorbance at 260 nm, and an appropriate volume of HU buffer was added (normalized to ribosome concentration).

### UBA purification and subsequent analysis of puromycylation

Overnight cultured cells were inoculated into 50 mL of fresh media to an OD_600 nm_ of 0.05 and incubated at 30 °C. At OD ~ 0.6, 4-NQO and MMS were added and cells incubated for an additional 30 min. Cells were harvested by centrifugation and washed three times with wash buffer (20 mM Tris pH 7.5, 140 mM KCl, 5 mM MgCl_2_ and 10 mM NEM). Cell pellets were flash frozen and stored at −80 °C. Frozen cell pellets were resuspended in 200 μL of lysis buffer (20 mM Tris pH 8.0, 140 mM KCl, 5 mM MgCl_2_, 200 μg/mL heparin, 5 mM NEM, 0.5 mM DTT, and protease inhibitor complete (ThermoFisher)). Cells were lysed using glass beads using a FastPrep and the resulting lysate clarified twice by centrifugation at 20,000 × *g* for 10 min at 4 °C. Absorbance was measured at 260 nm to estimate total RNA concentration. A total of 500 μg was diluted to 100 μL in lysis buffer, puromycin added to a final concentration of 2 mM and the reaction was incubated at 30 °C for 1 hr. Following the incubation, a 10-μL aliquot was added to 10 μL of HU buffer for input. The remaining volume was added to 20 μL UBA beads (BostonBiochem, #AM-130), which were prewashed with 20 mM Tris-HCl pH 7.5, 5 mM NEM, 0.1% NP40. The total volume was adjusted to 1 mL with the prewash buffer and lysates were incubated with beads for 1 h at 4 °C. The beads were washed 5 × with wash buffer (25 mM Tris, pH 7.5, 150 mM NaCl, 0.2% NP40) followed by collection over a spin column in wash buffer without detergent. 50 μL of HU buffer was added, heated briefly before loading on 8 or 10% SDS–PAGE gels. After transfer, western-blotting on the input and UBA-bound samples was conducted as described above.

### Fluorescence microscopy

Yeast cells expressing Hsp104-mCherry or Dcp2-GFP were fixed with 4% paraformaldehyde post treatment for 15 min. Cells were then centrifuged and resuspended in 0.1 M KPO_4_ pH 7.5, 1.2 M sorbitol, and sonicated briefly prior to mounting for fluorescence microscopy. Samples were imaged on a Nikon Eclipse E600 upright microscope at × 60 using OpenLab software (Improvision) through a Retiga EX charge-coupled-device camera (Q-Imaging). Image quantification was done using ImageJ software.

### Yeast-sensitivity assay

Yeast cells in biological triplicates were back-diluted from overnight culture and were treated with various damaging agents at mid-log-phase (OD_600_ of 0.5–0.7) for 30 min^88^. The cells were then washed twice and resuspended in YPD media to a final density of OD 0.8. In all, 5 μL of cell suspensions were added to 195 μl of YPD in 96-well non-treated polystyrene microplates, in technical repeats. The plates were incubated at 30 °C with shaking, and cell density was monitored every 10 min over 24–48 h at 600 nm on a microplate scanning spectrophotometer (Biotek PowerWave XS2).

Spot assays were performed using cells cultured to mid-log phase, treated with damaging agents for 30 min at 30 °C, washed twice and resuspended to OD ~ 0.8. Threefold dilutions were then spotted on YPD plates and incubated for 2–3 days at 30 °C.

### Peptide preparation for mass spec analysis

Yeast cell lysis and peptide digestion was performed as follows. Specifically, 3 L of mid-log cells were treated with various chemicals for 30 min at 30 °C. Cells were then pelleted, washed and resuspended in 5 mL of lysis buffer (20 mM Tris-HCl pH 7.5, 140 mM KCl, 10 mM MgCl_2_, 0.5 mM DTT, 5 mM NEM, 1 mM PMSF). Cells were lysed by FastPrep-24 with glass beads (top speed for 1 min with 1 min resting on ice for 5 cycles). Debris was pelleted at 15,000 × *g* for 10 min at 4 °C and the lysates were clarified twice at 30,000 × *g* for 20 min at 4 °C. The ribosome fraction from the lysate was enriched twice by centrifuging over a sucrose cushion (1.1 M sucrose, 20 mM Tris-HCl pH 7.5, 500 mM NH_4_Cl, 0.5 mM EDTA, 10 mM MgCl_2_, 5 mM NEM, 1 mM PMSF) at 107,100 × *g* for 16 hr at 4 °C in a Ti-45 rotor. The polysome-enriched fraction was resuspended in 10 mL RNase-free water and then incubated with 0.1 M NaOH at 37 °C for 30 min. Protein concentration was determined by Bradford assay. Proteins were precipitated with 10% trichloroacetic acid (TCA) at 4 °C for 16 hr. Samples were then centrifuged at 10,000 × *g* for 15 min at 4 °C. Pellets were washed with cold acetone and then resuspended in 8 M urea, 5 mM NEM, 1 mM PMSF. The samples were reduced for 1 h with 1/25 volume of 200 mM DTT in 25 mM NH_4_HCO_3_. Alkylation was performed by the addition of 200 mM chloroacetamide in 25 mM NH_4_HCO_3_ for 1 h at room temperature (RT) in the dark. The samples were then quenched by adding the same volume of reducing agent as the chloroacetamide and incubated at RT for 5 min. The urea concentration was adjusted to 1 M using 25 mM NH_4_HCO_3_, pH > 8. Proteins were digested first by LysC protease (Wako Chemicals USA, #NC9223464) at a protein ratio of 1:250 (w/w) for 4 hr at 37 °C. Lysate were further digested using Trypsin (Pierce Trypsin Protease #90057) at a protein ratio of 1:50 (w/w) at 37 °C for 16 hr. The digestion was quenched by adding trifluoroacetic acid to a final concentration of 1%. The precipitate in the sample was removed by centrifuging at 3000 × *g* for 5 min. The peptides were desalted by Sep-Pak C18 purification. Pre-enrichment samples were collected at the end of the elution step. Peptides were then dried.

### K-ε-GG peptide immunoprecipitation for LC-MS/MS

K-ε-GG peptide immunoprecipitation was performed using the PTMScan Ubiquitin-Remnant Motif (K-ε-GG) Kit (#5562, Cell Signaling Technology) as per the standard protocol. Following the final elution of the protocol, peptides were cleaned using Millipore Ziptips (Millipore Sigma #Z720070), dried and stored at −20 °C until LC-MS analysis.

### Tandem mass spectrometry analysis

The sample was resuspended in 17 µl 5% acetonitrile, 0.1% formic acid. Nano-scale LC separation of tryptic peptides was performed on a Dionex Ultimate 3000 Rapid Separation LC system (Thermo Scientific). Of the digests, 4 µl was loaded onto a 20 μl nanoViper sample loop (Thermo Scientific), and separated on a C18 analytical column (Acclaim PepMap RSLC C18 column, 2 μm particle size, 100 Å pore size, 75 µm × 25 cm (Thermo Scientific) by the application of either a linear-2-hour gradient from 2% to 32% acetonitrile in 0.1% formic acid for the analysis of the ribosome-enriched samples and a linear-2-hour gradient from 4% to 36% acetonitrile for the analysis of subsequent glygly enrichment, with a column flow rate set to 250 nL/min.

Analysis of the eluted tryptic peptides was performed online using a Q Exactive Plus mass spectrometer (Thermo Scientific) possessing a Nanospray Flex Ion source (Thermo Scientific) fitted with a stainless steel nano-bore emitter operated in positive electro-spray ionization mode at a capillary voltage of 1.9 kV. Settings that differed for detecting glygly enriched peptides are shown in brackets. Data-dependent acquisition of full MS scans within a mass range of 380–1500 m/z at a resolution of 70,000 was performed, with the automatic gain control (AGC) target set to 3 × 10^6^, and the maximum fill time set to 200 ms. High-energy collision-induced dissociation fragmentation of the top 15 (8) most intense peaks was performed with a normalized collision energy of 28, with an intensity threshold of 4 × 10^4^ (1.3 × 10^4^) counts and an isolation window of 3.0 m/z, excluding precursors that had an unassigned, +1, +7, or +8 charge state. MS/MS scans were conducted at a resolution of 17,500, with an AGC target of 2 × 10^5^ and a maximum fill time of 100 ms (300 ms). Dynamic exclusion was performed with a repeat count of 2 and an exclusion duration of 30 s, whereas the minimum MS ion count for triggering MS/MS was set to 4 × 10^3^ counts. The resulting MS/MS spectra were analyzed using Proteome Discoverer software (version 2.0.0.802, Thermo Scientific), which was set up to search the *S. cerevisiae* proteome database, as downloaded from www.uniprot.org/proteomes (ID number UP000002311). Peptides were assigned using SEQUEST HT^89^, with search parameters set to assume the digestion enzyme trypsin with a maximum of two missed cleavage, a minimum peptide length of 6, precursor mass tolerances of 10 ppm, and fragment mass tolerances of 0.02 Da. Carbamidomethylation of cysteine was specified as a static modification, whereas glygly on lysine, oxidation of methionine, and N-terminal acetylation were specified as dynamic modifications. The target false discovery rate of 0.01 (strict) was used as validation for peptide-spectral matches (PSMs) and peptides. Proteins that contained similar peptides and which could not be differentiated based on the MS/MS analysis alone were grouped, to satisfy the principles of parsimony. Label-free quantification as previously described^90^ was performed in Proteome Discoverer with a minimum Quan value threshold of 0.0001 using unique peptides, and “3 Top N” peptides used for area calculation. All samples were analyzed in triplicate, and the resulting values were averaged.

### Polysomes analysis, hel2 3 × FLAG cross-linking to ribosomes

Yeast cultures were grown to mid-log phase before addition of either cycloheximide (to a final concentration of 100 μg/mL, 3 μg /mL, or 0.015 μg/mL), or 0.1% MMS, or 5 μg/mL 4-NQO for 30 min. Cultures were chilled on ice and formaldehyde added to 1% of the culture volume. After 1 h, these were quenched by addition of 1/10 volume of 2.5 M glycine in 25 mM Tris-base. Cells were pelleted by centrifugation at 4 °C, resuspended in polysome-lysis buffer (20 mM Tris pH 7.5, 140 mM KCl, 5 mM MgCl_2_, 0.5 mM DTT, 1% Triton-100, 100 μg/mL cycloheximide, 200 μg/mL heparin), washed once and lysed with glass beads using a FastPrep (MP Biomedical). Supernatant from cleared lysate corresponding to 1 mg of total RNA was layered over a 10–50% sucrose gradient and centrifuged at 37,000 rpm for 160 min in an SW41Ti (Beckman) swinging bucket rotor. Gradients were fractionated using a Brandel tube-piercing system combined with continuous absorbance reading at A_254 nm_. Protein was extracted by the NaOH/TCA method and resuspended in HU buffer (8 M Urea, 5% SDS, 200 mM Tris pH 6.8, 100 mM DTT).

### Reporters for measuring mRNA decay in mammalian-cell culture

Control mRNAs were prepared using runoff transcription on PCR-amplified DNA templates and T7 RNA polymerase^91^, followed by PAGE purification. 8-oxo-G-mRNAs were generated by ligating an RNA oligo containing the modified guanosine to a similarly in vitro-transcribed RNA. To eliminate heterogeneity at the ligation site, the 3′-end contained a self-cleaving hammerhead ribozyme sequence, which results in a 2′–3′-cyclic phosphate after cleavage. The ends were dephosphorylated using T4 polynucleotide kinase (NEB) in 100 mM morpholinoethanesulfonate (MOPS)-NaOH (pH 5.5), 10 mM MgCl_2_, 10 mM β-mercaptoethanol, 300 mM NaCl^92^ leaving a 3′-OH group; the reaction was incubated at 37 °C for 5 h, extracted with phenol/chloroform and ethanol precipitated.

An RNA oligo with a modified guanosine was purchased from Chemgenes (5′-GGACUACAAAGAC (8-oxo-rG) ACGACGACAAGUAAUCUCU). The 5′-end of the oligo was phosphorylated using T4 PNK (NEB) in the presence of ATP followed by ligation to the 3′-end of the mRNA using T4 RNA ligase 2 (NEB) and a DNA splint at a molar ratio of (1:1.5:1.2). The RNA and DNA were combined, heated to 80 °C, and slowly cooled to room temperature before addition of buffer and ligase followed by incubation at 37 °C overnight. The ligated products were purified on denaturing PAGE, eluted in 300 mM NaCl overnight and ethanol precipitated.

In order to observe turnover of the mRNA reporters in vivo, both the ligated RNA and transcribed RNA controls were capped using the *Vaccinia* capping system (NEB) in the presence of [α-^32^P]-GTP and SAM. This was followed by addition of a 3′-polyA sequence using *E. coli* polyA polymerase (NEB). Reactions were assembled per the manufacturer’s instructions and incubated for 30 min at 37 °C, phenol/chloroform extracted, and ethanol precipitated. Prior to electroporation, the RNAs were CIP treated (NEB) to remove 5′-phosphates, followed by phenol chloroform extraction and ethanol precipitation.

### Measurements of mRNA decay

TREx HEK cells (ThermoFisher Scientific) were cultured in DMEM (Gibco/life technologies) + FBS (Sigma) + Pen/Strep (Gibco/life technologies) using standard protocols. RNA was electroporated into cells using a Neon Transfection System (Thermo Fisher Scientific). On the day of experiment, cells were washed and resuspended in buffer R at ~1–2 × 10^7^ cells/mL. Approximately 5 μg of reporter mRNA and 5 μg of GFP mRNA were combined with 100 μL of cells/buffer for each reaction. After electroporation, cells were immediately added to 1 mL media without antibiotics and recovered for 30 min. The cells and media were then divided equally into six aliquots and incubated until reaching the indicated timepoints. RNA was isolated using Trizol and precipitated in isopropanol before analysis by northern blotting.

### Northern blotting

In all, 1 μg of RNA from each timepoint was resolved on 1.5% formaldehyde agarose gel, followed by transfer to positively charged nylon membrane (GE Lifesciences) using a vacuum blotter (Biorad). Nucleic acids were UV cross-linked to the membrane and baked at 80 °C for 15 min. Membranes were then pre-hybridized in Rapid-Hyb buffer (GE Lifesciences) for 30 min in a hybridization oven. Radiolabeled DNA probe, which was labeled using polynucleotide kinase and [γ-^32^P]-ATP, was added to the buffer and incubated overnight. Membranes were washed with nonstringent buffer (2 × SSC, 0.1% SDS) three times, in some cases followed by three washes in stringent buffer (0.2 × SSC, 0.1% SDS), all at hybridization temperature. Membranes were exposed to a phosphorimager screen and analyzed using a Biorad personal molecular imager.

Data were analyzed using Prism (Graphpad) and half-life calculated by fitting the data using linear regression to a one phase decay equation.

### Reporting summary

Further information on research design is available in the Nature Research Reporting Summary linked to this article.

## Supplementary information

Supplementary Information

Peer Review

Reporting Summary

## Data Availability

The mass spectrometry data, including the.msf and.xml files for the proteomics data, are available in the ProteomeXchange database under accession number PXD013834 within the PRIDE repository. The source data used to generate plots in Figs. 1a–e, 2b–d, 3g–k, and 6a; and Supplementary Figs. 1b–d, g–j, and 5 are provided as a Source Data file. Uncropped and unprocessed immunoblot scans for Figs. 4i–m and 5 are also provided as a Source Data File. All other data are available from the corresponding author on reasonable request.

## Source Data

Source Data
