# Supplementary material for: Oxidation and alkylation stresses activate ribosome-quality control
Source: Nat Commun. 2019 Dec 9;10:5611. doi: 10.1038/s41467-019-13579-3 (PMC6901537; doi:10.1038/s41467-019-13579-3)
Supplement: Supplementary file 1 — Supplementary Information [file 41467_2019_13579_MOESM1_ESM.pdf]

Supplementary Information

Oxidation and alkylation stresses activate ribosome-quality control

Liewei L. Yan, Carrie L. Simms, Fionn McLoughlin, Richard D. Vierstra and Hani S. Zaher*

Affiliations: Department of Biology, Washington University in St. Louis, St. Louis, MO, USA 63130.

***Correspondence to**: hzaher@wustl.edu

Department of Biology

Washington University in St. Louis

Campus Box 1137, One Brookings Drive

St. Louis, MO, USA 63130

Phone: (314) 935-7662

Fax: (314) 935-4432

Supplementary Table 1: Encrichment of DiGly peptides of ribosomal proteins upon 4-NQO treatment as assessed by MS

									Log2(enrichment)	
**Annotated Sequence**	**# Proteins**	**# PSMs**	**# Missed Cle**	**Other**	**Description**	**Master Protein Accessions**			**Biological repeat 1**	**Biological repeat 2**
[R].GPRNTEEKGWVPVTK.[L]	1	26	2	s	uS5-40S ribosomal protein S2 OS=Saccharomyces cerevisiae (s	P25443, K33	uS2, K33	P25443, K33	3.9658	9.0267
[K].ALPDAVTIIEPKEEEPILAPSVK.[D]	1	12	1	s	uS3-40S ribosomal protein S3 OS=Saccharomyces cerevisiae (s	P05750, K21	uS3, K212	P05750, K212	6.1297	4.8073
[R].ELEKKFPDR.[H]	1	23	2	s	eS7-40S ribosomal protein S7-B OS=Saccharomyces cerevisiae	P48164, K83	eS7B, K83	P48164, K83	7.0334	2.8423
[K].EKVEEQEQQQQQIIK.[I]	1	41	1	s	uS10-40S ribosomal protein S20 OS=Saccharomyces cerevisiae	P38701, K8	uS10, K8	P38701, K8	7.1711	2.3567
[K].IDLASGKITDFIK.[F]	1	6	1	s	eS4-40S ribosomal protein S4-B OS=Saccharomyces cerevisiae	P0CX36, K16	eS4B, K168	P0CX36, K168	-3.7694	1.5236
[R].GMTSKPAGFMKK.[L]	1	34	1	s	uS19-40S ribosomal protein S15 OS=Saccharomyces cerevisiae	Q01855, K52	uS19, K52/K	Q01855, K52	0.3543	1.1619
[R].GMTSKPAGFMKK.[L]	1	7	1	s	uS19-40S ribosomal protein S15 OS=Saccharomyces cerevisiae	Q01855, K52	uS19, K52	Q01855, K52	-2.8939	0.2508
[R].EKLAEVYKAEK.[D]	1	22	2	s	eS24-40S ribosomal protein S24-B OS=Saccharomyces cerevisi	P0CX32, K43	eS24B, K43	P0CX32, K99	-0.2725	-0.108
[K].ALQSLTSKGYVK.[T]	2	11	1	s	eS10-40S ribosomal protein S10-B OS=Saccharomyces cerevisi	P46784, K52	eS10B, K52	P46784, K31	0.5305	-0.4071
[K].DFNQAKHEEIDTK.[N]	2	10	1	s	eS10-40S ribosomal protein S10-B OS=Saccharomyces cerevisi	P46784, K31	eS10B, K31	P46784, K31	0.1359	-0.848
[R].AAKLAAPENEKPAPVR.[T]	1	32	1	s	uS19-40S ribosomal protein S15 OS=Saccharomyces cerevisiae	Q01855, K64	uS19, K64	Q01855, K52	-0.1751	-1.3964
[R].VTPTKTEVIIR.[A]	1	11	1	s	uS3-40S ribosomal protein S3 OS=Saccharomyces cerevisiae (s	P05750, K45	uS3, K45	P05750, K45	-0.206	-1.4999
[K].VAPAPFGAKSTK.[S]	2	7	1	l	eL8-60S ribosomal protein L8-A/B OS=Saccharomyces cerevisi	P29453, K15	eL8B, K15	P29453, K15	2.5913	3.6707
[R].GFTLAEVKAAGLTAAYAR.[T]	2	18	1	l	eL13-60S ribosomal protein L13-B OS=Saccharomyces cerevisi	P40212, K81	eL13B, K81	P40212, K81	-0.6688	1.7433
[R].EAGEVKDDGAFVK.[F]	1	6	1	l	uL16-60S ribosomal protein L10 OS=Saccharomyces cerevisiae	P41805, 191	uL16, 191	P41805, 191	0.451	0.7936
[K].KAEYFAKLR.[E]	1	23	2	l	uL10-60S acidic ribosomal protein P0 OS=Saccharomyces cerev	P05317, K14	uL10, K14	P05317, K14	-1.2239	0.2176
[R].YGATSTNPAKSASAR.[G]	2	6	1	l	uL22-60S ribosomal protein L17-A/B OS=Saccharomyces cerev	P05740, K13	uL22B, K13	P05740, K13	0.0396	-0.2854
[K].SVKFVQGLLQNAAANAEAK.[G]	2	7	1	l	uL22-60S ribosomal protein L17-A/B OS=Saccharomyces cerev	P46990, P05	uL22A/B, K8	P46990, P05740,	2.7084	-0.5265
[R].APKGQNTLILR.[G]	1	18	1	l	eL18-60S ribosomal protein L18-A OS=Saccharomyces cerevisi	P0CX49, K13	eL18A, K133	P0CX49, K133	-1.6306	-0.9053
[K].DGAKFIEVR.[N]	2	24	1	l	uL6-60S ribosomal protein L9-A OS=Saccharomyces cerevisiae	P05738, K11	uL6A, K110	P05738, K110	-1.8218	-2.2574
[R].IFASNEVIAKSR.[Y]	1	7	1	l	eL20-60S ribosomal protein L20-B OS=Saccharomyces cerevisi	P0CX24, K38	eL20B, K38	P0CX24, K38	-2.1037	-2.5634
[K].WAASSWAKK.[I]	2	24	1	l	eL14-60S ribosomal protein L14-A OS=Saccharomyces cerevisi	P36105, K10	eL14A, K101	P36105, K101	-0.0283	-2.614
[K].VLIDGPKAGVPR.[Q]	2	8	1	l	eL14-60S ribosomal protein L14-A OS=Saccharomyces cerevisi	P36105, K50	eL14A, K50	P36105, K50	-1.9594	-3.8129
[K].SGKYTLGYKSTVK.[S]	1	19	2	l	eL30-60S ribosomal protein L30 OS=Saccharomyces cerevisiae	P14120, K22	eL30, K22	P14120, K22	-1.3121	-4.95
[K].VIEQPITSETAMKK.[V]	1	36	1	l	uL23-60S ribosomal protein L25 OS=Saccharomyces cerevisiae	P04456, K73	uL23, K73	P04456, K73	-1.689	-5.4107
[R].TLSDYNIQKESTLHLVLR.[L]	3	70	1	u	eS31-Ubiquitin-40S ribosomal protein S31 OS=Saccharomyces	P05759, K63	eS31, K63	P05759, K63	3.8474	3.2548
[R].LIFAGKQLEDGR.[T]	3	66	1	u	eS31-Ubiquitin-40S ribosomal protein S31 OS=Saccharomyces	P05759, K48	eS31, K48	P05759, K48	1.5975	0.4546
[K].SKIQDKEGIPPDQQR.[L]	3	146	2	u	eS31-Ubiquitin-40S ribosomal protein S31 OS=Saccharomyces	P0CH09, K29,	eS31, K29/K	P0CH09, K29/K33	-0.7686	-0.3536

Supplementary Table 2: Reporter sequences

Name	mRNA sequence	Predicted protein product
Control	G AAC UUU AAG AAG GAG AUA UAC AU **AUG** GCA UAC CCA UAC GAC GUC CCA GAC UAC GCU AUG UCU AAG AUU AAA GGU GAC GUU AAG UGG UUU GAC GAG UCC AAA GGA UUC GGU UUC AUU ACU CCG GAA GAC GGC AGC AAA GAC GUG UUC GUA CAC UUC UCU GCA AUC CAG ACU GAC GGU UUU AAA ACU CUU GCU GAA GGU CAG CGC GUA GAG UUC GAA AUC ACU GAC GGU GCC AAA GGC CCU UCU GCU GCA AAA GUA AUC GCU CUG AAA UCG GAC UAC AAA GAC GAC GAC GAC AAG **UAA** UCU CU	**M**AYPYDVPDYAMSKIKGDVKWFDESKG FGFITPEDGSKDVFVHFSAIQTDGFKT LAEGQRVEFEITDGAKGPSAAKVIALK SDYKDDDDK-S
8oxo-ORF	G AAC UUU AAG AAG GAG AUA UAC AU **AUG** GCA UAC CCA UAC GAC GUC CCA GAC UAC GCU AUG UCU AAG AUU AAA GGU GAC GUU AAG UGG UUU GAC GAG UCC AAA GGA UUC GGU UUC AUU ACU CCG GAA GAC GGC AGC AAA GAC GUG UUC GUA CAC UUC UCU GCA AUC CAG ACU GAC GGU UUU AAA ACU CUU GCU GAA GGU CAG CGC GUA GAG UUC GAA AUC ACU GAC GGU GCC AAA GGC CCU UCU GCU GCA AAA GUA AUC GCU CUG AAA UCG GAC UAC AAA GAC **8oxo-G** AC GAC GAC AAG **UAA** UCU CU	**M**AYPYDVPDYAMSKIKGDVKWFDESKG FGFITPEDGSKDVFVHFSAIQTDGFKT LAEGQRVEFEITDGAKGPSAAKVIALK SDYKD**X***DDK*-S

8oxo-UTR	G AAC UUU AAG AAG GAG AUA UAC AU **AUG** GCA UAC CCA UAC<br>GAC GUC CCA GAC UAC GCU AUG UCU AAG AUU AAA GGU GAC<br>GUU AAG UGG UUU GAC GAG UCC AAA GGA UUC GGU UUC AUU<br>ACU CCG GAA GAC GGC AGC AAA GAC GUG UUC GUA CAC UUC<br>UCU GCA AUC CAG ACU GAC GGU UUU AAA ACU CUU GCU GAA<br>GGU CAG CGC GUA GAG UUC GAA AUC ACU GAC GGU GCC AAA<br>GGC CCU UCU GCU GCA AAA GUA AUC GCU CUG UAA UCG GAC<br>UAC AAA GAC **8oxo-G** AC GAC GAC AAG **UAA** UCU CU	**M**AYPYDVPDYAMSKIKGDVKWFDESKG<br>FGFITPEDGSKDVFVHFSAIQTDGFKT<br>LAEGQRVEFEITDGAKGPSAAKVIAL-<br>SDYKD**X**DDDK-S
Stop	G AAC UUU AAG AAG GAG AUA UAC AU **AUG** GCA UAC CCA UAC<br>GAC GUC CCA GAC UAC GCU AUG UCU AAG AUU AAA GGU GAC<br>GUU AAG UGG UUU GAC GAG UCC AAA GGA UUC GGU UUC AUU<br>ACU CCG GAA GAC GGC AGC AAA GAC GUG UUC GUA CAC UUC<br>UCU GCA AUC CAG ACU GAC GGU UUU AAA ACU CUU GCU GAA<br>GGU CAG CGC GUA GAG UUC GAA AUC ACU GAC GGU GCC AAA<br>GGC CCU UCU GCU GCA AAA GUA AUC GCU CUG AAA UCG GAC<br>UAC AAA GAC **UAA** GAC GAC AAG **UAA** UCU CU	**M**AYPYDVPDYAMSKIKGDVKWFDESKG<br>FGFITPEDGSKDVFVHFSAIQTDGFKT<br>LAEGQRVEFEITDGAKGPSAAKVIALK<br>SDYKD-DDK-S
no stop	GGGG AAC UUU AAG AAG GAG AUA UAC AU **AUG** GCA UAC CCA<br>UAC GAC GUC CCA GAC UAC GCU AUG UCU AAG AUU AAA GGU<br>GAC GUU AAG UGG UUU GAC GAG UCC AAA GGA UUC GGU UUC<br>AUU ACU CCG GAA GAC GGC AGC AAA GAC GUG UUC GUA CAC<br>UUC UCU GCA AUC CAG ACU GAC GGU UUU AAA ACU CUU GCU<br>GAA GGU CAG CGC GUA GAG UUC GAA AUC ACU GAC GGU GCC<br>AAA GGC CCU UCU GCU GCA AAA GUA AUC GCU CUG AAA UC	**M**AYPYDVPDYAMSKIKGDVKWFDESKG<br>FGFITPEDGSKDVFVHFSAIQTDGFKT<br>LAEGQRVEFEITDGAKGPSAAKVIALK

Supplementary Table 3: Yeast strains

Strain	Genotype	Source
BY4741 (matA)	*MATa (his3Δ1 leu2Δ0 met15Δ0 ura3Δ0)*	Dharmacon
DOM34Δ	*BY4741; dom34::KanMX*	Dharmacon
XRN1Δ	*BY4741; xrn1::KanMX*	Dharmacon
SKI2Δ	*BY4741; ski2::KanMX*	Dharmacon
LTN1Δ	*BY4741; ltn1::KanMX*	Dharmacon
HEL2Δ	*BY4741; hel2::LEU2*	This study
uS3(R116A,R117A)	*BY4741; rps3 (R116A, R117A)-HIS3*	Simms et al., 2018
cdc33-ts4-2	*BY4741; cdc33-ts4-2-HIS3*	This study
cdc48	*MATa (his3-11,-15 leu2-3 met15Δ0 ura3-1, trp1-1)*	Deshales lab
cdc48; LTN1Δ	*MATa; ltn1::KanMX*	Deshales lab
HEL2-3xFLAG	*MATa; HEL2-3xFLAG-HIS3*	This study
uS3-FLAG	*MATa; uS3-FLAG-HIS3*	This study
uS4-FLAG	*MATa; uS4-FLAG-HIS3*	This study
uS5-FLAG	*MATa; uS5-FLAG-HIS3*	This study
uS7-FLAG	*MATa; uS7-FLAG-HIS3*	This study
uS10-FLAG	*MATa; uS10-FLAG-HIS3*	This study
uS12-FLAG	*MATa; uS12-FLAG-HIS3*	This study
uS17-FLAG	*MATa; uS17-FLAG-HIS3*	This study
uS24-FLAG	*MATa; uS24-FLAG-HIS3*	This study
uS28-FLAG	*MATa; uS28-FLAG-HIS3*	This study
uS31-FLAG	*MATa; uS31-FLAG-HIS3*	This study
uL5-FLAG	*MATa; uL31-FLAG-HIS3*	This study
HEL2Δ; uS7-FLAG	*MATa; hel2::LEU2; uS7-FLAG-HIS3*	This study
HEL2Δ; uS10-FLAG	*MATa; hel2::LEU2; uS10-FLAG-HIS3*	This study
HSP104-mCherry	*MATa; HSP104-mCherry-HIS3*	This study
LTN1Δ; HSP104-mCherry	*MATa; ltn1::KanMx; HSP104-mCherry-HIS3*	This study
LTN1Δ; SSA1-mCherry	*MATa; ltn1::KanMx; SSA1-mCherry-HIS3*	This study
LTN1Δ; SSA3-mCherry	*MATa; ltn1::KanMx; SSA3-mCherry-HIS3*	This study
DCP2-GFP; HSP104-mCherry	*MATa; pDCP2-GFP-URA3; HSP104-mCherry-HIS3*	This study
LTN1Δ; DCP2-GFP; HSP104-mCherry	*MATa; ltn1::KanMx; pDCP2-GFP-URA3; HSP104-mCherry-HIS3*	This study
HEL2Δ; pAG-HEL2	*MATa; hel2::LEU2; pAG-426-HEL2*	This study
HEL2Δ; pAG-Empty	*MATa; hel2::LEU2; pAG-426*	This study

Supplementary Table 4: Plasmids

Plasmid name	Description	Source
pRP1315	DCP2-GFP-URA3	Nissan and Parker, *Methods in Enzymology* , 2008
pAG-426-gpd-ccdb	Empty plasmid for Hel2	Lindquist lab, Addgene. Alberti et al., *Yeast* , 2007
pAG-HEL2	pAG-426-Hel2	This study

Supplementary Table 5: Primer sequences

Oligo Name	Sequence	Used for
RNA oligo-FLAG	G GAC UAC AAA GAC (8-oxo-rG)AC GAC GAC AAG UAA UCU CU	RNA oligo ligated to '8oxo-ORF' RNA and '8oxo-UTR' RNA
5' all	AGA TCT CCC AGA ATT CAT TAA TAC GAC TCA CTA TAG	5' end of all mRNAs
splint 1	AGA GAT TAC TTG TCG TCG TCG TCT TTG TAG TCC GAT TTC AGA GCG ATT ACT TTT GC	3' end control mRNA, ligation splint
oxoG HH 3'	GCT CTG AAA TTT CGT CCT CAC GGA CTC ATC AGA CGA GCT AGC TCG TGA TTT CAG AGC GAT TAC TTT TGC	3' end of 8oxo-ORF mRNA fragment
oxoG HH UTR 3'	GCT CTG AAA TTT CGT CCT CAC GGA CTC ATC AGA CGA GCT AGC TCG T GA **TTA** CAG AGC GAT TAC TTT TGC	3' end of 8oxo-UTR mRNA fragment
HH UTR splint	AGA GAT TAC TTG TCG TCG TCG TCT TTG TAG TCC GAT TAC AGA GCG ATT ACT TTT GC	Splint for 8oxo-UTR ligation
3' stop	TTA GTC TTT GTA GTC CGA TTT CAG AGC GAT TAC TTT TGC	3' end with Stop codon at 8oxo-G position
hs18S-probe	CCA AGA ATT TCA CCT CTA GCG GCG	Northern blotting
hsGAPDH-probe	GTC ATC ATA TTT GGC AGG TTT TTC TAG ACG GCA GGT CAG GTC CAC CAC TGA CAC GTT GGC	Northern blotting
GFP-probe	CGT AGC CTT CGG GCA TGG CGG ACT TGA AGA AGT CGT GCT GCT TCA TGT GGT CGG GGT AGC	Northern blotting
Hel2-FLAG-FW	AAG AAA GGC AAA CAA AAG CAG CTG TTA TTC CAC ATT GGT GTA GGG GGA GGC GGG GGT GGA	Tagging Hel2 with FLAG
Hel2-FLAG-REV	TTA AGA CTT TCA TTT CTC TAA TGC TAT TGT CAG TTA CAG GGA ATT CGA GCT CGT TTA AAC	Tagging Hel2 with FLAG
Hsp104-mCherry-FW	AAT CAC GAA GCT ACT ATA GGG GCT GAC ACG TTA GGT GAT GAC GAT AAT GAG GAC AGT ATG	Tagging Hsp104 with mCherry
Hsp104-mCherry-REV	AAA CAA ATT CCA TTA GTT TTA TTA ATT ATA TAT ATT ATA TTA CTG ATT CTT GTT CGA AAG	Tagging Hsp104 with mCherry
Ssa3-mCherry-FW	GGA GGT GGA GAA GAT ACA GGT CCA ACA GTG GAA GAG GTT GAT GGG GGA GGC GGG GGT GGA	Tagging Ssa3 with mCherry
Ssa3-mCherry-REV	GGT TAA ACA TAA AAA GTA GCT AAA TAG AAC ACT ATA GAA GGA ATT CGA GCT CGT TTA AAC	Tagging Ssa3 with mCherry
Ssa1-mCherry-FW	CCA GCT CCA GAG GCT GAA GGT CCA ACC GTT GAA GAA GTT GAT GGG GGA GGC GGG GGT GGA	Tagging Ssa1 with mCherry
Ssa1-mCherry-REV	ATC ATT AAA AGA CAT TTT CGT TAT TAT CAA TTG CCG CAC CGA ATT CGA GCT CGT TTA AAC	Tagging Ssa1 with mCherry
uS3-FLAG-FW	AAA CTG AAG CTC AAG CTG AAC CAG TTG AAG CTG GGG GAG GCG GGG GTG GA	Tagging uS3 with FLAG
uS3-FLAG-REV	ATA GTT TAT TTA TGT ATT TAA TAA TTA AAT GAA TTC GAG CTC GTT TAA AC	Tagging uS3 with FLAG
uS7-FLAG-FW	AAG AAG AAG GAT GAA TTG GAA CGT GTT GCC AAG TCT AAC CGT GAA CGC AAA TGC TGC GTG	Tagging uS7 with FLAG
uS7-FLAG-REV	AAA AAC ATA TGT AAT ATT GAA AAT CTT TCA CTT TTT TTA GGA GCT CGT TTT CGA CAC TGG	Tagging uS7 with FLAG
eS7-FLAG-FW	ACT GGT AAG CAA ATT GTT TTC GAA ATT CCA AGT GAA ACT CAT GGG GGA GGC GGG GGT GGA	Tagging eS7 with FLAG
eS7-FLAG-REV	GTT GTT ACT AAC TAA TAA TAA AGA TTT AGA TAT TAT TAT AGA ATT CGA GCT CGT TTA AAC	Tagging eS7 with FLAG
uS4-FLAG-FW	GAA GCT TCC GGT GAA GCT GCT GAA GAA GCC GAA GAC GAA GAA GGG GGA GGC GGG GGT GGA	Tagging uS4 with FLAG
uS4-FLAG-REV	GAA AAG TTA TAA AAT TTA TTA TAC AGT TAT GAA ATC AGC GGA ATT CGA GCT CGT TTA AAC	Tagging uS4 with FLAG
eS10-FLAG-FW	ATT CAA GAA AGA AAC CCA ACT CAA AGA CCA CAA AGA AGA TAT GGG GGA GGC GGG GGT GGA	Tagging eS10 with FLAG
eS10-FLAG-REV	AGA AGA ATG TTA CAA AAG AGA AAA TTC AAT TGG AGC TAC GGA ATT CGA GCT CGT TTA AAC	Tagging eS10 with FLAG
eS12-FLAG-FW	ACT GAT GAA TTG TCC ATG ATC ATG GAA CAC TTC TCC CAA CAA GGG GGA GGC GGG GGT GGA	Tagging eS12 with FLAG
eS12-FLAG-REV	TAT GTG ATC TAA CTC TAG AAA AAG TAC TAA TGA ACA TCA CGA ATT CGA GCT CGT TTA AAC	Tagging eS12 with FLAG
eS17-FLAG-FW	AAC GTT TCT GCC CAA AGA GAC AGA CGT TAC AGA AAG AGA GTT GGG GGA GGC GGG GGT GGA	Tagging eS17 with FLAG
eS17-FLAG-REV	AAT ATT TTG AAT AGT AAA TTA TTT TAA ATA GCT TTC TAA TGA ATT CGA GCT CGT TTA AAC	Tagging eS17 with FLAG
eS24-FLAG-FW	GGT AAG AGA TTG GCT AAG AAG GTT GCT CGT CGT AAC GCC GAT GGG GGA GGC GGG GGT GGA	Tagging eS24 with FLAG
eS24-FLAG-REV	CTA AAC AAT CCG TAA AAA TGA TTT AAC GTT TTG AAG TTA GGA ATT CGA GCT CGT TTA AAC	Tagging eS24 with FLAG
eS28-FLAG-FW	TTG GTT CTA ATG GAA TCT GAA CGT GAA GCT CGT CGT TTG CGT GGG GGA GGC GGG GGT GGA	Tagging eS28 with FLAG

eS28-FLAG-REV	TAG ATT TTG TAT AGC TGC AAC CTT CAA TCT GCA AAT AAG CGA ATT CGA GCT CGT TTA AAC	Tagging eS28 with FLAG
eS31-FLAG-FW	TTG TAC TGT GGT AAG TGT CAT TCC GTC TAC AAG GTT AAC GCT GGG GGA GGC GGG GGT GGA	Tagging eS31 with FLAG
eS31-FLAG-REV	AAT TTA ACG ATC AAT TAA AAT ATA TAA GTT TTA AAA ATA CGA ATT CGA GCT CGT TTA AAC	Tagging eS31 with FLAG
uL5-FLAG-FW	TCT TGG TTC AAG CAA AAG TAC GAT GCT GAT GTT TTG GAC AAA GGG GGA GGC GGG GGT GGA	Tagging uL5 with FLAG
uL5-FLAG-REV	TAT GAG TAT ATA AAT GTA TTA TAT TTC TTC ACA AAC TAA TGA ATT CGA GCT CGT TTA AAC	Tagging uL5 with FLAG
eL15-FLAG-FW	TGG AAG AGA CAA AAC ACT TTG TCC TTG TGG AGA TAC AGA AAA GGG GGA GGC GGG GGT GGA	Tagging eL15 with FLAG
eL15-FLAG-REV	AAA CAA AAG TTT GCC CAA TAA AAT TAT ATT TTC CAT CAA CGA ATT CGA GCT CGT TTA AAC	Tagging eL15 with FLAG

Yan et al., Supplementary Fig. 1

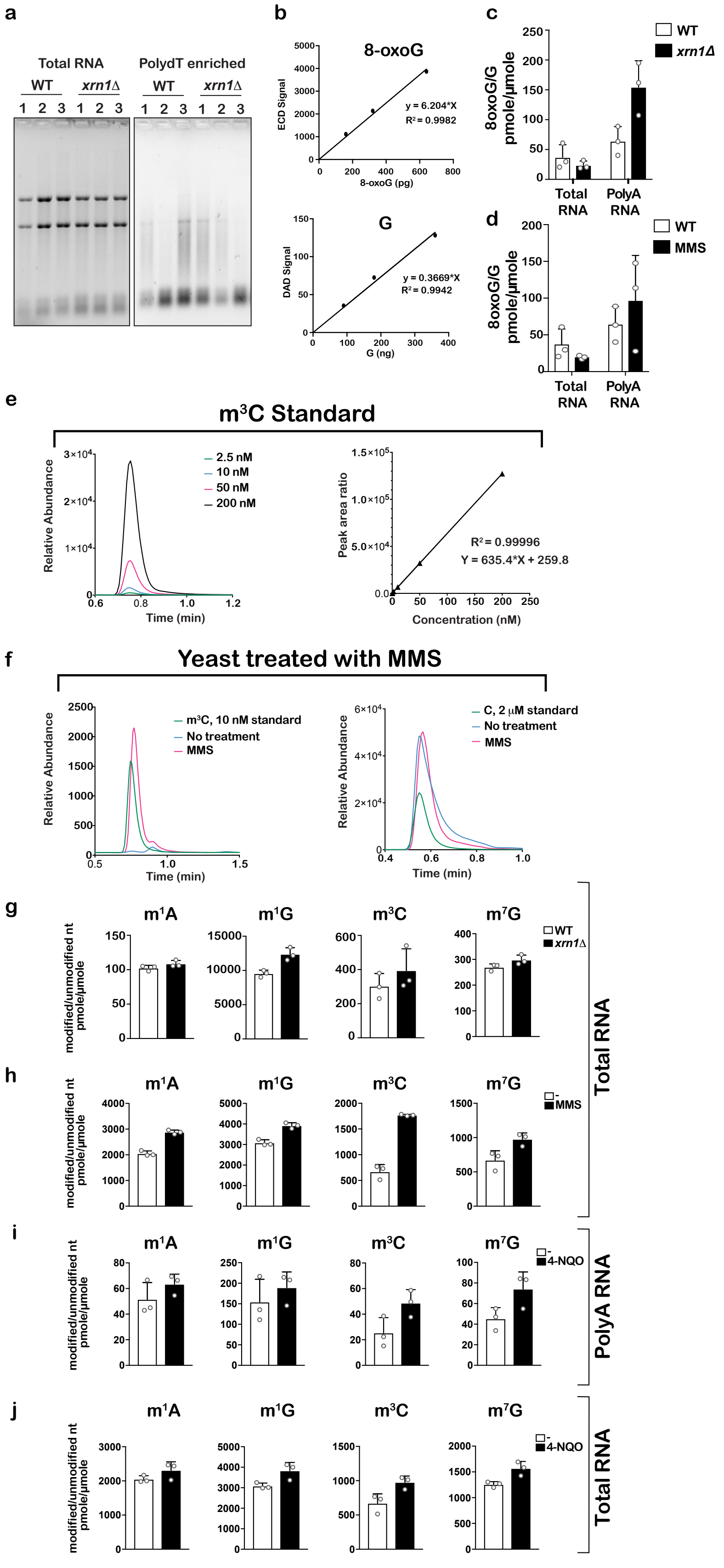

Supplementary Fig. 1| Quantification of modified nucleotides using LC-MS. a, Formaldehyde-agarose electrophoresis of total RNA and polyA RNA isolated from the indicated strains and stained with ethidium bromide. Shown are the samples from three biological triplicates. **b,** Standard-calibration curves for 8-oxoG (MRM signal) and G (Diode-array signal at 254 nm). **c-d,** Bar graph showing the quantification results of 8-oxoG in the indicated samples using LC-MS. **e,** Right are selected overlaid ion chromatograms for m^3C standards at the indicated concentrations. Left is the standard-calibration curve with values integrated from the chromatograms shown to the right. **f**, Representative ion chromatograms for m^3C (right) and DAD chromatograms for C (left) from samples isolated from yeast grown in the absence and presence of MMS. In both cases, standard chromatograms were overlaid on top to confirm the identity of the nucleoside. **g-j,** Bar graph showing the relative amount of the indicated modified bases to the corresponding unmodified ones: in total RNA in the presence/absence of Xrn1 (**g**), total RNA in the absence/presence of MMS (**h**), and in polyA (**i**) and total RNA (**j**) in the absence/presence of 4-NQO. In all cases, bars represent the average (±SD) of at least three independent biological replicates. Source data are provided as a Source Data file.

Yan et al., Supplementary Fig. 2

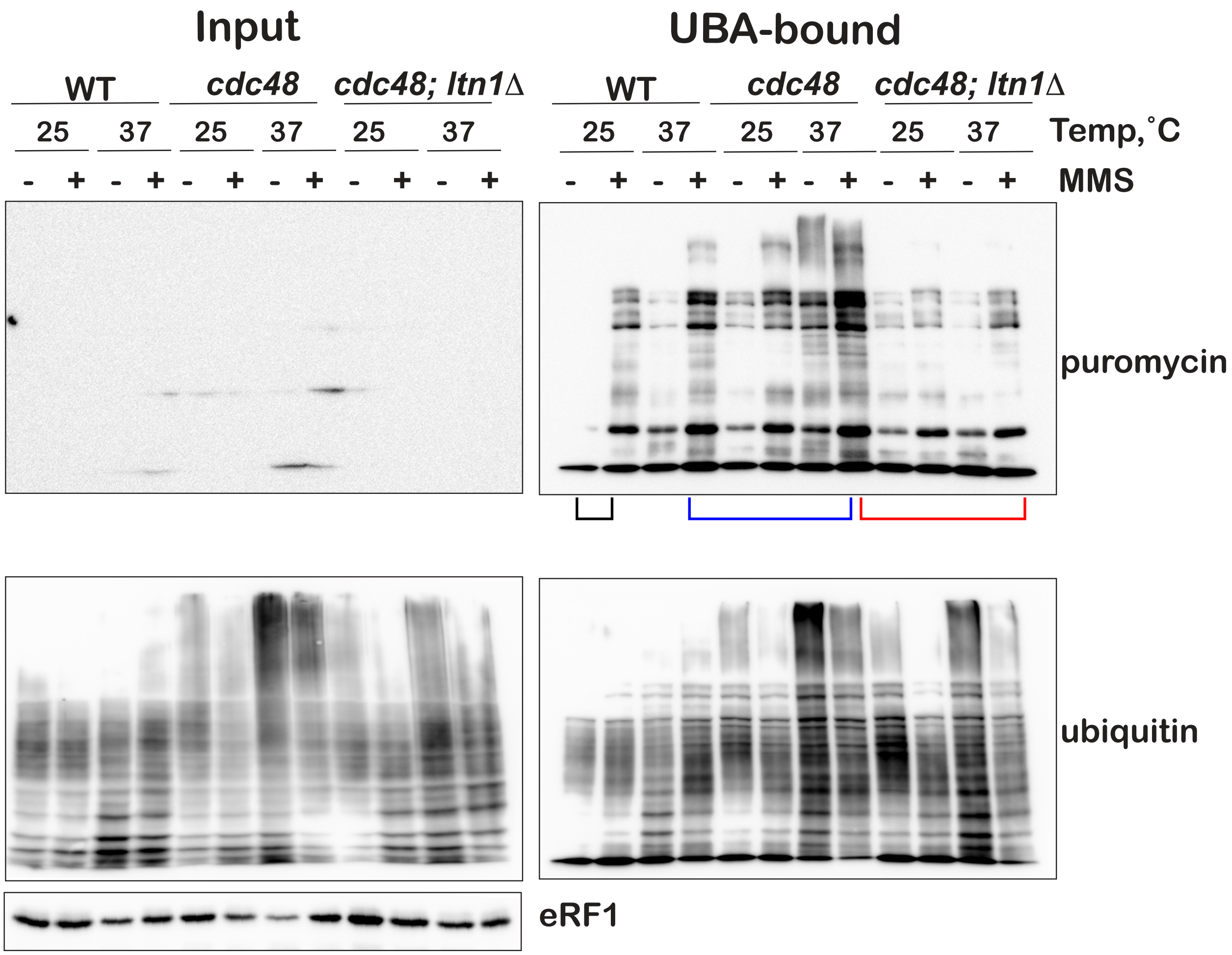

Supplementary Fig. 2| Ubiquitinated-nascent peptides accumulate in the presence of the alkylating agent MMS. Similar to the western-blotting analysis shown in Fig. 2b, but in the presence of MMS instead of 4-NQO.

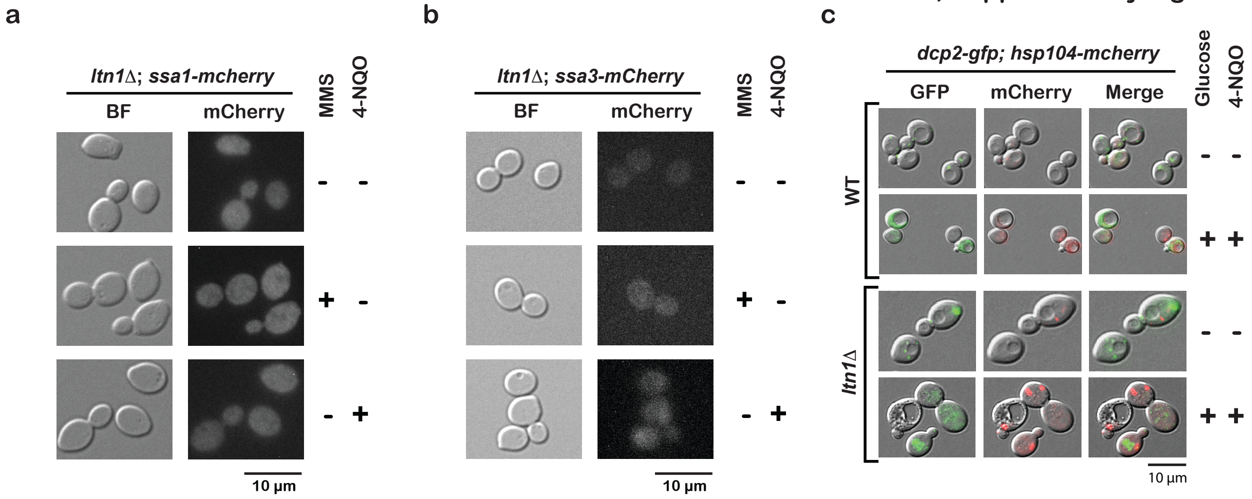

Supplementary Fig. 3| Ssa1- and Ssa3-mCherry proteins do not form foci in the presence of 4-NQO, and Hsp104-mCherry foci are distinct from P bodies. a-b, Brightfield- and fluorescence-microscope images of yeast cells with endogenously tagged Ssa1 (**a**) and Ssa3 (**b**) proteins in the absence of Ltn1. Cells were treated as indicated. **c,** mCherry- and GFP-fluorescence images of Hsp104-mCherry yeast cells overexpressing Dcp2-GFP. Cells were treated as indicated and mCherry and GFP signals were overlaid. Glucose deprivation was used to induce P-bodies formation, seen as green foci in the images. Scale bar is 10 μm.

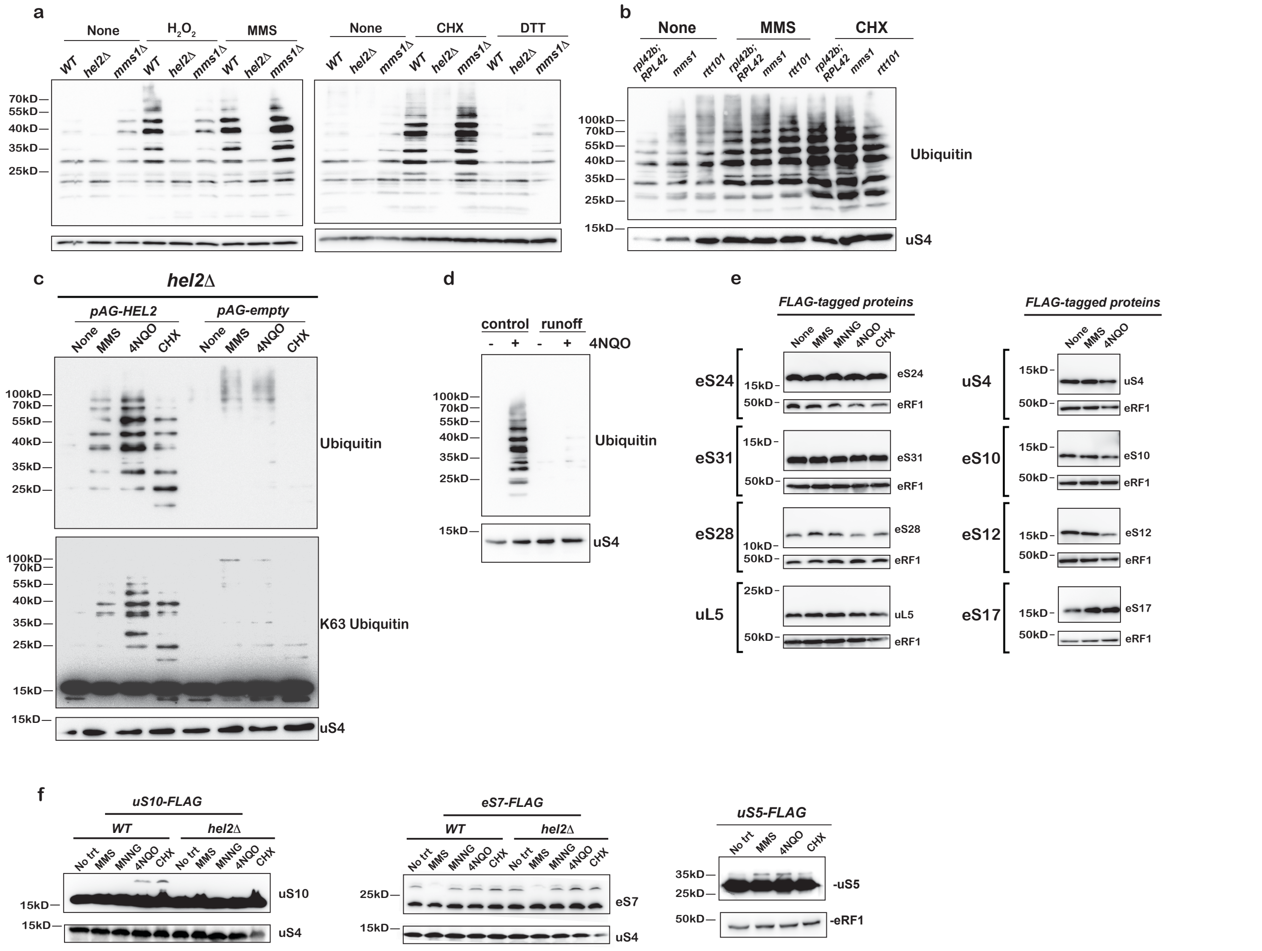

Supplementary Fig. 4| Ribosomal-proteins ubiquitination in the presence of damaging agents requires Hel2 and active translation. a-b, Western-blot analysis showing that only the deletion of *HEL2,* but neither that of *MMS1* nor *RTT101* results in inhibition of ribosomal-proteins ubiquitination in the presence of the indicated chemical agents. **c,** Western-blot analysis showing that plasmid-borne *HEL2* rescues the loss of ribosomal-protein phenotype seen in the presence of alkylating and oxidizing agents, but an empty plasmid does not. The middle membrane was probed with an anti-K63-linked-ubiquitin antibody. **d,** Western-blot analysis used to show that translation runoff (through glucose deprivation) results in loss of ribosomal-proteins ubiquitination upon the addition of 4-NQO. **e-f,** Western-blot analysis with FLAG antibody used to follow the ubiquitination of the depicted ribosomal proteins in the presence of the indicated compounds.

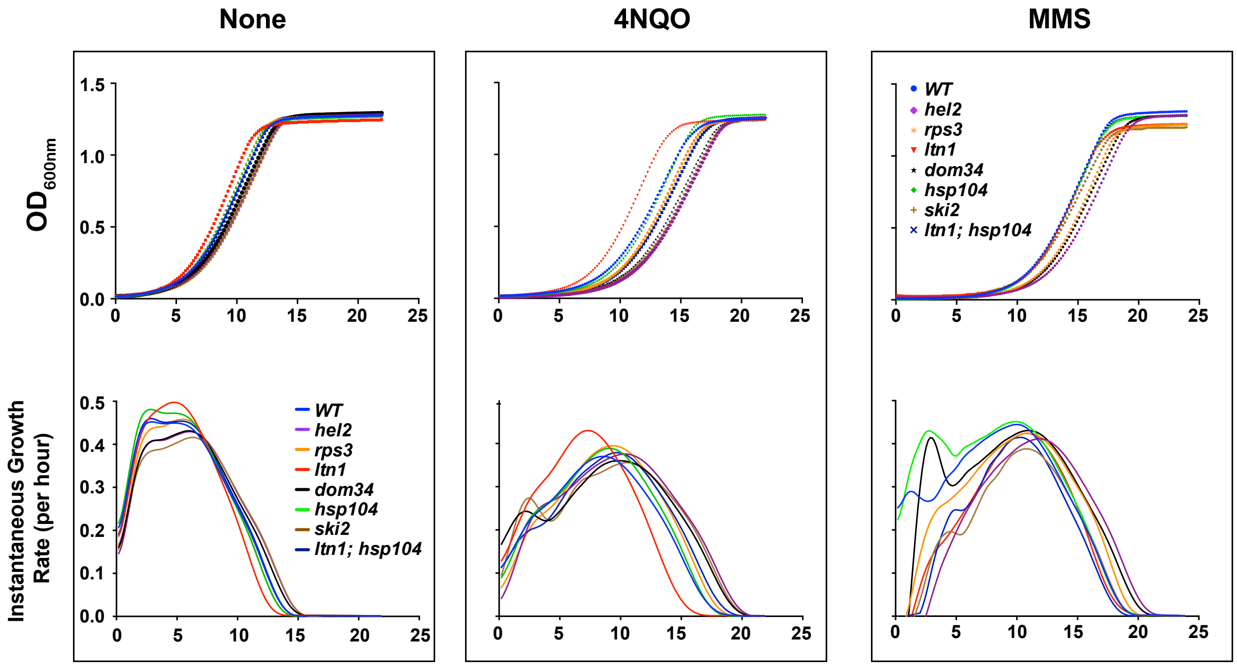

Supplementary Fig. 5| Mutations in NGD and RQC components render cells sensitive to 4-NQO and MMS addition. Similar to what is shown in Figure 6, but with an expanded set of factors. Top is a plot of OD_{600nm} over time of the indicated strains after a mock treatment or a 30-minute challenge with 4-NQQ (1 μg/mL) or MMS (0.1 % MMS). Bottom is a plot of the first derivative of the data on top to calculate the instantaneous growth rate and the corresponding lag time needed to reach the maximum rate. Data was collected in technical duplicates from three biological replicates.

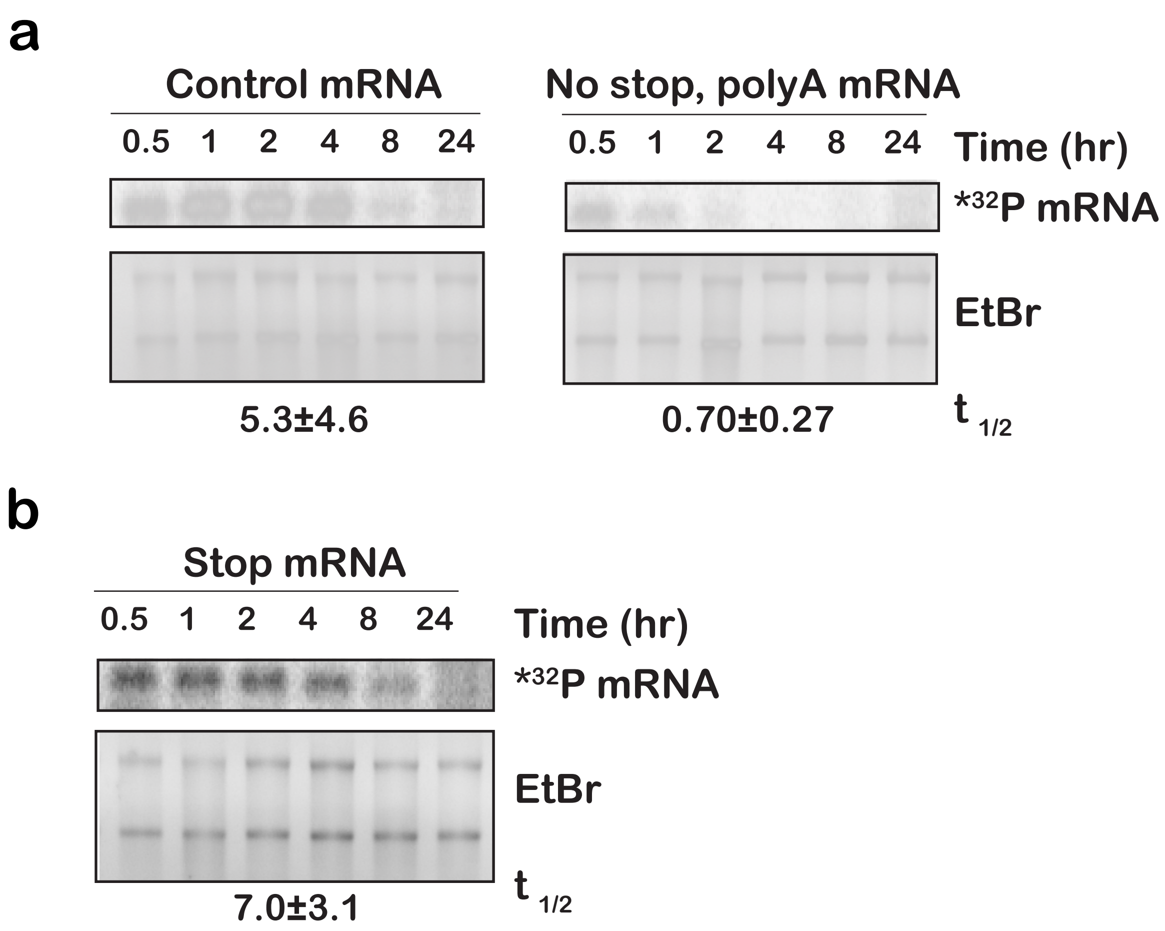

Supplementary Fig. 6| In-vitro-generated non-stop mRNA reporter decays much faster relative to a control reporter in HEK293 cells. a-b, Phosphorimager scans used to follow the turnover of the indicated radiolabeled mRNA reporters post electroporation into HEK293 cells. Stop mRNA indicates a reporter where a stop codon was inserted in the place of 8-oxoG in the 8-oxoG-ORF reporter (Fig. 7). The half lives were determined from duplicates (± SD).
