# Supplementary material for: Oxidation and alkylation stresses activate ribosome-quality control
Source: Nat Commun. 2019 Dec 9;10:5611. doi: 10.1038/s41467-019-13579-3 (PMC6901537; doi:10.1038/s41467-019-13579-3)
Supplement: Supplementary file 3 — Reporting Summary [file 41467_2019_13579_MOESM3_ESM.pdf]

nature research

Corresponding author(s): Hani S Zaher

Last updated by author(s): Oct 4, 2019

Reporting Summary

Nature Research wishes to improve the reproducibility of the work that we publish. This form provides structure for consistency and transparency in reporting. For further information on Nature Research policies, see Authors & Referees and the Editorial Policy Checklist.

Statistics

For all statistical analyses, confirm that the following items are present in the figure legend, table legend, main text, or Methods section.

n/a	Confirmed	
☐	☒	The exact sample size (*n*) for each experimental group/condition, given as a discrete number and unit of measurement
☐	☒	A statement on whether measurements were taken from distinct samples or whether the same sample was measured repeatedly
☐	☒	The statistical test(s) used AND whether they are one- or two-sided<br>*Only common tests should be described solely by name; describe more complex techniques in the Methods section.*
☒	☐	A description of all covariates tested
☒	☐	A description of any assumptions or corrections, such as tests of normality and adjustment for multiple comparisons
☐	☒	A full description of the statistical parameters including central tendency (e.g. means) or other basic estimates (e.g. regression coefficient) AND variation (e.g. standard deviation) or associated estimates of uncertainty (e.g. confidence intervals)
☐	☒	For null hypothesis testing, the test statistic (e.g. *F*, *t*, *r*) with confidence intervals, effect sizes, degrees of freedom and *P* value noted<br>*Give P values as exact values whenever suitable.*
☒	☐	For Bayesian analysis, information on the choice of priors and Markov chain Monte Carlo settings
☒	☐	For hierarchical and complex designs, identification of the appropriate level for tests and full reporting of outcomes
☒	☐	Estimates of effect sizes (e.g. Cohen's *d*, Pearson's *r*), indicating how they were calculated

Our web collection on statistics for biologists contains articles on many of the points above.

Software and code

Policy information about availability of computer code

Data collection	These are indicated in the manuscript where applicable.<br>6400 Series Triple Quadrupole B.08.02, Image Quant LAS 4000, QCapture64x version 2.0.13, Agilent ChemStation for LC 3D systems B.03.02, Improvision Openlab 5.5.0, Gen5 3.04.17, Proteome DiscovererTM software version 2.0.0.802
Data analysis	Agilent Masshunter Qualitative Analysis Navigator b.08.00, GraphPad Prism 7.0d, ImageQuant TL, Microsoft Excel 16.27, ImageJ 1.52b, Perseus documentation 1.6.5.0, Quantity One 4.6.6

For manuscripts utilizing custom algorithms or software that are central to the research but not yet described in published literature, software must be made available to editors/reviewers. We strongly encourage code deposition in a community repository (e.g. GitHub). See the Nature Research guidelines for submitting code & software for further information.

Data

Policy information about availability of data

All manuscripts must include a data availability statement. This statement should provide the following information, where applicable:

- Accession codes, unique identifiers, or web links for publicly available datasets
- A list of figures that have associated raw data
- A description of any restrictions on data availability

The tandem MS sequence, .msf and .xml files for the proteomics data are available in the ProteomeXchange database under accession number PXD013834 within the PRIDE repository (http://www.proteomcexchange.org./).

Field-specific reporting

Please select the one below that is the best fit for your research. If you are not sure, read the appropriate sections before making your selection.

☒ Life sciences ☐ Behavioural & social sciences ☐ Ecological, evolutionary & environmental sciences

For a reference copy of the document with all sections, see nature.com/documents/nr-reporting-summary-flat.pdf

Life sciences study design

All studies must disclose on these points even when the disclosure is negative.

Sample size	These are indicated in the figure legends and/or methods<br>For determination of the amount of modified nucleotides, we used 3 RNA preparations from three different yeast cultures.<br>For foci calculations, we used three different biological repeats. For each sample, more than 120 cells were counted.<br>For growth assays, three biological repeats were used and the growth of each was measured in duplicates.<br>For proteomics, biological duplicates were used.
Data exclusions	For analysis of mRNA half life, one timepoint from one biological replicate was excluded as the sample was lost during processing.
Replication	Western analysis was repeated on different days with different samples to ensure reproducibility. Northern analysis and determination of mRNA half life values reported are biological duplicates from independent cell transfections.
Randomization	Yeast strains were assigned to their groups based on their genotypes, which was confirmed using selective plating and PCR genotyping.
Blinding	Foci determination was blinded, for which the folder names were randomly assigned by a person not involved in the project before counting was done.

Reporting for specific materials, systems and methods

We require information from authors about some types of materials, experimental systems and methods used in many studies. Here, indicate whether each material, system or method listed is relevant to your study. If you are not sure if a list item applies to your research, read the appropriate section before selecting a response.

Materials & experimental systems

n/a	Involved in the study	
☐	☒	Antibodies
☐	☒	Eukaryotic cell lines
☒	☐	Palaeontology
☒	☐	Animals and other organisms
☒	☐	Human research participants
☒	☐	Clinical data

Methods

n/a	Involved in the study	
☒	☐	ChIP-seq
☒	☐	Flow cytometry
☒	☐	MRI-based neuroimaging

Antibodies

Antibodies used	These are included in the methods.<br>Ubiquitin was detected by a mouse monoclonal antibody conjugated to HRP (Santa Cruz, P4D1; 1:2,000 v/v dilution). Flag-tagged proteins were detected by a mouse monoclonal antibody (Sigma; 1:5,000 v/v dilution). Puromycin was detected by a mouse monoclonal antibody (Millipore, #MABE343; 1:5,000 v/v dilution). uS4 (Rps9) was detected by a rabbit monoclonal antibody (Abcam, 1:5,000 v/v dilution). Phospho-Histone H2A.X was detected by a rabbit monoclonal antibody (Cellsignal, 1:3,000 v/v dilution). K48-linked-ubiquitin chains were detected by anti-ubiquitin (Cellsignal, 1:3,000 v/v dilution, linkage-specific k48). K63-linked-ubiquitin chains were detected by (Cellsignal, 1:3,000 v/v dilution, linkage-specific k63). eRF1 was detected by a polyclonal rabbit antibody81. Secondary antibodies of rabbit anti mouse IgG (Thermo Scientific) and goat anti rabbit IgG (Thermo Scientific) were used at (1:10,000 v/v dilution).
Validation	All antibodies were used for western analysis and sizes of the bands matched the predicted sizes of the proteins.<br>Antibodies for tags like FLAG and HA were validated by probing samples from yeast that do not have tagged proteins. Puromycin antibody was validated using samples that were not treated with puromycin. uS4 antibody was validated using samples that were enriched for ribosomes as well as polysomes. Phospho-Histone H2A.X was validated using samples that were not treated with DNA-damaging agents. Secondary antibodies were validated using blots that were not probed with the appropriate primary anitbody.

Eukaryotic cell lines

Policy information about cell lines

Cell line source(s)	TREx HEK cells (ThermoFisher Scientific)
Authentication	Cell lines were obtained from a commercial vendor and were not authenticated.
Mycoplasma contamination	Cells were not tested for mycoplasma contamination.
Commonly misidentified lines (See ICLAC register)	Not applicable
