# Supplementary material for: Oxidation and alkylation stresses activate ribosome-quality control
Source: Nat Commun. 2019 Dec 9;10:5611. doi: 10.1038/s41467-019-13579-3 (PMC6901537; doi:10.1038/s41467-019-13579-3)
Supplement: Supplementary file 2 — Peer Review [file 41467_2019_13579_MOESM2_ESM.pdf]

Editorial Note: This manuscript has been previously reviewed at another journal that is not operating a transparent peer review scheme. This document only contains reviewer comments and rebuttal letters for versions considered at Nature Communications.
